# Taxonomic revision of *Epipactis* Zinn (Orchidaceae) on the Korean Peninsula

**DOI:** 10.3897/phytokeys.278.195337

**Published:** 2026-08-21

**Authors:** Jung-Sim Lee, Jee-Yeon Koo, Min-Ha Kim

**Affiliations:** 1 Korean Peninsula Plant Diversity Institute, Yongin 17095, Republic of Korea National Institute of Biological Resources, Species Diversity Research Division Incheon Republic of Korea; 2 The Korean Society of Botanical Illustrators, Seoul, Republic of Korea Korean Peninsula Plant Diversity Institute Yongin Republic of Korea; 3 National Institute of Biological Resources, Species Diversity Research Division, Incheon 22689, Republic of Korea The Korean Society of Botanical Illustrators Seoul Republic of Korea

**Keywords:** *
Epipactis
*, *Epipactis xanthophaea*, Korean Peninsula, Orchidaceae, taxonomy

## Abstract

The genus *Epipactis* (Orchidaceae) is widely distributed across Europe, Asia, tropical Africa, and North America. In Korea, only *Epipactis thunbergii* and *Epipactis papillosa* have traditionally been recognized, and the taxonomy of the genus has not been critically reassessed. Based on examination of protologues, herbarium specimens, and field observations, *Epipactis xanthophaea*, previously misidentified as *E. thunbergii*, is confirmed to occur in Korea and is recognized as a distinct species. Accordingly, three species of *Epipactis* (*E. thunbergii*, *E. xanthophaea*, and *E. papillosa*) are recognized on the Korean Peninsula. Their diagnostic characters and distribution ranges are clarified, and a revised taxonomic treatment is provided.

## Introduction

*Epipactis* Zinn (Orchidaceae, Epidendroideae, Neottieae) is widely distributed across temperate to subalpine regions of Europe and Asia, and also occurs in tropical Africa and North America (Pridgeon et al. 2005; Chen et al. 2009). The genus is divided into two sections, *Arthrochilium* Irmisch and *Epipactis* s.str., primarily based on lip morphology (Fateryga and Fateryga 2018a). Most species belong to sect. *Epipactis*, whereas approximately 10–12 species are placed in sect. *Arthrochilium* (Efimov 2004; Fateryga and Fateryga 2018a; Pedersen et al. 2018). The number of accepted species in *Epipactis* varies widely depending on taxonomic concepts and treatments, ranging from approximately 15 to 79 species (Pridgeon et al. 2005; Chen et al. 2009; Pedersen et al. 2018; POWO 2025; WFO 2025). On the Korean Peninsula, *Epipactis* has traditionally been represented by two species, *Epipactis thunbergii* A. Gray and *Epipactis papillosa* Franch. & Sav. (National Institute of Biological Resources 2019; Korea National Arboretum 2021). *Epipactis thunbergii* belongs to sect. *Arthrochilium*, whereas *E. papillosa* is assigned to sect. *Epipactis*. Although several studies have been conducted in Korea (Chung and Chung 2007, 2015; Chung 2009; Han et al. 2013; Chung et al. 2018), taxonomic treatments based on detailed comparative morphology remain limited. *Epipactis xanthophaea* Schltr. was originally described from northeastern China and belongs to sect. *Arthrochilium*. The species closely resembles *E. thunbergii* in general habit and floral morphology and has therefore frequently been confused with that species in East Asia. However, it differs from E. thunbergii in several floral characters, particularly in lip structure, as described in the protologue (Schlechter 1922).

Recent field surveys revealed the presence of a taxon closely resembling *E. thunbergii* in the northern and southwestern parts of the Baekdudaegan mountain range and on islands along the western coast. Due to its strong morphological similarity, this taxon has long been treated as conspecific with *E. thunbergii*. However, reliable diagnostic information for its identification remains insufficient in existing floristic accounts (Kim and Lee 1997; Lee 2011; National Institute of Biological Resources 2019; Korea National Arboretum 2021). These observations indicate that the taxonomic identity and species limits of Korean *Epipactis* have not been critically reassessed based on detailed morphological evidence.

This study aims to clarify the taxonomic identity of a taxon previously misidentified as *E. thunbergii*, to confirm its conspecificity with *E. xanthophaea*, and to document its occurrence in Korea. Based on comparative morphological analysis, this study recognizes three *Epipactis* species on the Korean Peninsula (*E. thunbergii*, *E. xanthophaea*, and *E. papillosa*), clarifies their diagnostic characters and distribution ranges, and provides a revised taxonomic treatment, including typification where necessary.

## Materials and methods

### Material examined

To clarify the taxonomic identities of *Epipactis thunbergii* and *Epipactis papillosa* on the Korean Peninsula, the original type specimens deposited at the Harvard University Herbaria (GH) and the Muséum National d’Histoire Naturelle (MNHN), respectively, were examined based on high-resolution digital images. In addition, type material of *Epipactis xanthophaea* was examined based on high-resolution digital images. Given the close morphological similarity between *E. thunbergii* and *E. xanthophaea*, extensive voucher material from A, AMES, HNWP, IFP, K, KB, KH, KUN, KYO, LE, MHA, MW, PE, TI, TNS, and WU was examined (Thiers 2023). Furthermore, morphological data on *E. xanthophaea* were obtained from both field observations and digital examination of specimens from Korea and northeastern China, supplemented by additional digital examination of herbarium specimens from across its distribution range (Suppl. material 1).

Specimens housed in TI were examined during multiple visits (2017, 2018, 2023), including historical collections from the Korean Peninsula. Specimens deposited in LE were verified during a visit in 2019, and detailed observations were conducted using high-resolution digital images. Additional materials from KYO and TNS (2017) were also directly observed. Japanese specimens were examined to evaluate morphological continuity between the Korean Peninsula and continental Northeast Asia.

For several type specimens that could not be directly examined, high-resolution digital images were used for comparison, and such cases are explicitly indicated in the taxonomic treatment. Taxonomic decisions, including synonymization, were based on direct comparison with type material whenever available. Nomenclatural information from IPNI (2025) was used only as a reference for name verification and not as a primary source for taxonomic decisions. Limitations associated with the use of digital images were acknowledged and carefully considered in the interpretation of morphological characters.

### Distribution analysis

Distribution patterns of *Epipactis* species on the Korean Peninsula were analyzed based on specimen data obtained through field surveys. Distribution data from China, Japan, and Russia were compiled from relevant floristic literature and online databases, including Flora of China (Chen et al. 2009), Flora of Japan (Inoue 2016), Trudy Imperatorskago S.-Peterburgskago Botaniceskago Sada. Acta Horti Petropolitani (Komarov 1901), Flora of Russia (Komarov 1935), Plantarium Online Plant Identification Guide (Plantarium 2025), Global Biodiversity Information Facility (GBIF 2025), and Plants of the World Online (POWO 2025).

### Morphological analysis

To compare the morphological characteristics of the three species, we analyzed their original protologues (Gray 1856; Franchet and Savatier 1878; Schlechter 1922), relevant taxonomic literature (Efimov 2004; Averyanova 2013), and fresh materials collected throughout Korea. Morphological measurements of *E. thunbergii*, *E. xanthophaea*, and *E. papillosa* were obtained from living specimens in the field using a digital vernier caliper, based on detailed field notes. Photographic documentation and comparative character tables were also prepared.

Field observations of the *E. thunbergii*-like taxon in Korea have been conducted since 2012 and continued through 2025. Additional investigations in northeastern China between 2013 and 2019 confirmed the occurrence of morphologically identical populations, supporting its broader distribution beyond the Korean Peninsula. Direct field investigation in Russia was not conducted; Russian materials were evaluated based on herbarium specimens, particularly those deposited in LE, and available digital resources. All voucher specimens cited in this study are deposited at KB.

## Results and discussion

**Figure 1.**
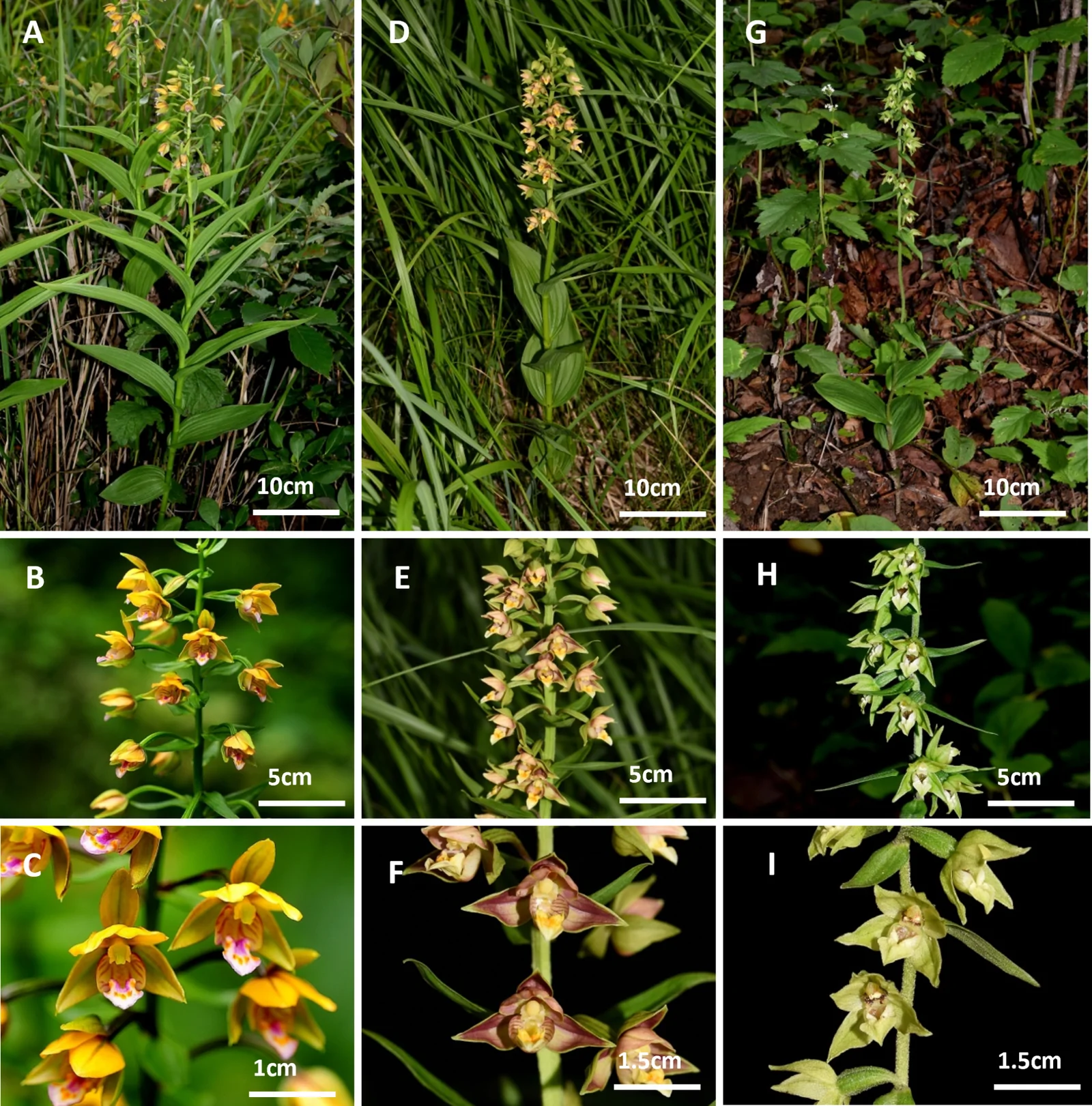
Wild habitat photographs of three *Epipactis* species in Korea. **A–C**. *E. thunbergii*; **D–F**. *E. xanthophaea*; **G–I**. *E. papillosa*. Photos by J.S. Lee.

**Figure 2.**
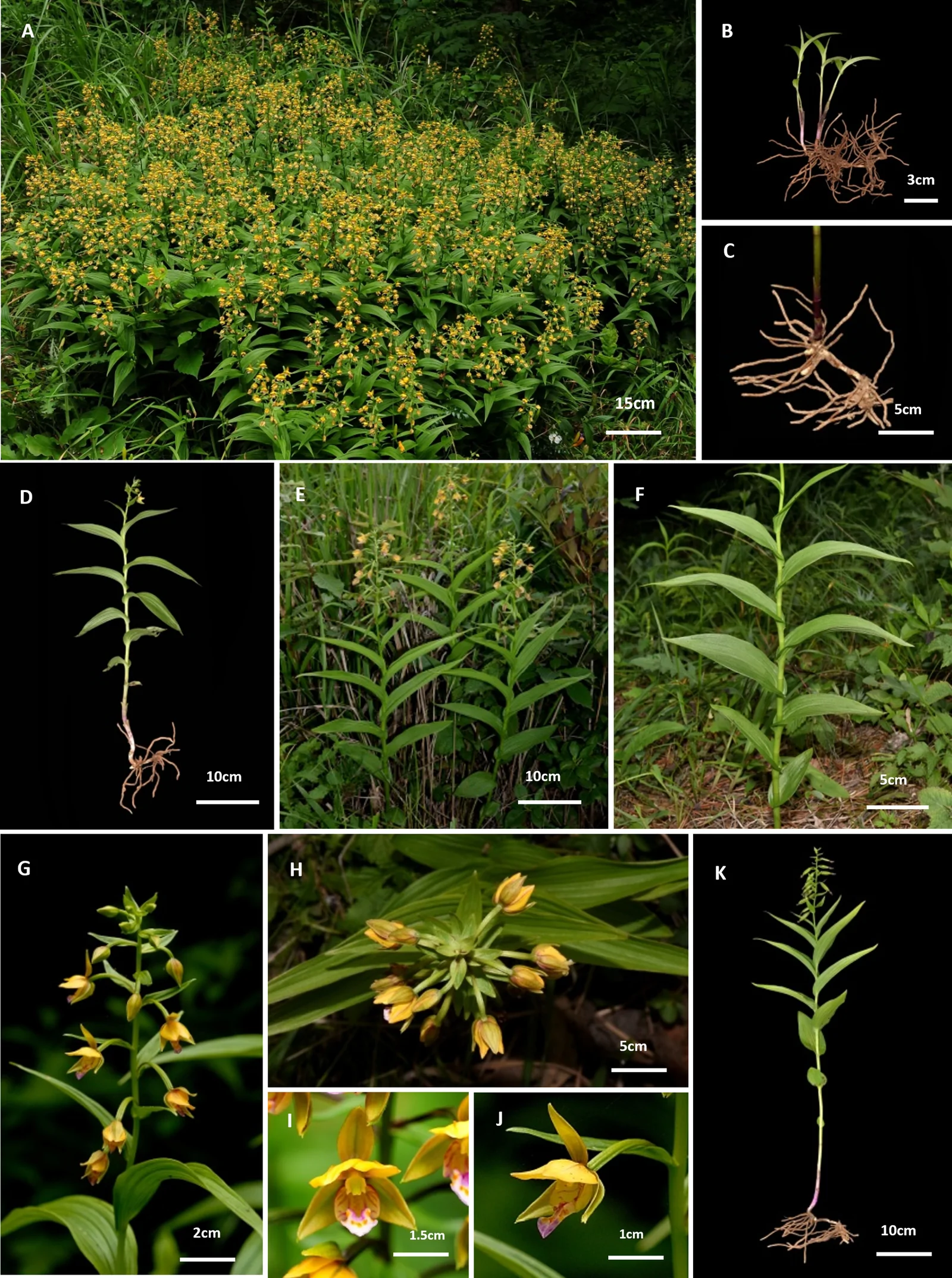
Photographs of *Epipactis thunbergii* from Korea. **A**. Habit; **B**. Young plants; **C**. Rhizome and roots; **D**. Flowering plant; **E**. Plants in flower; **F**. Leaves; **G**. Inflorescence; **H**. Top view of inflorescence; **I**. Front view of flower; **J**. Side view of flower; **K**. Fruiting plant. Photos by J.S. Lee.

**Figure 3.**
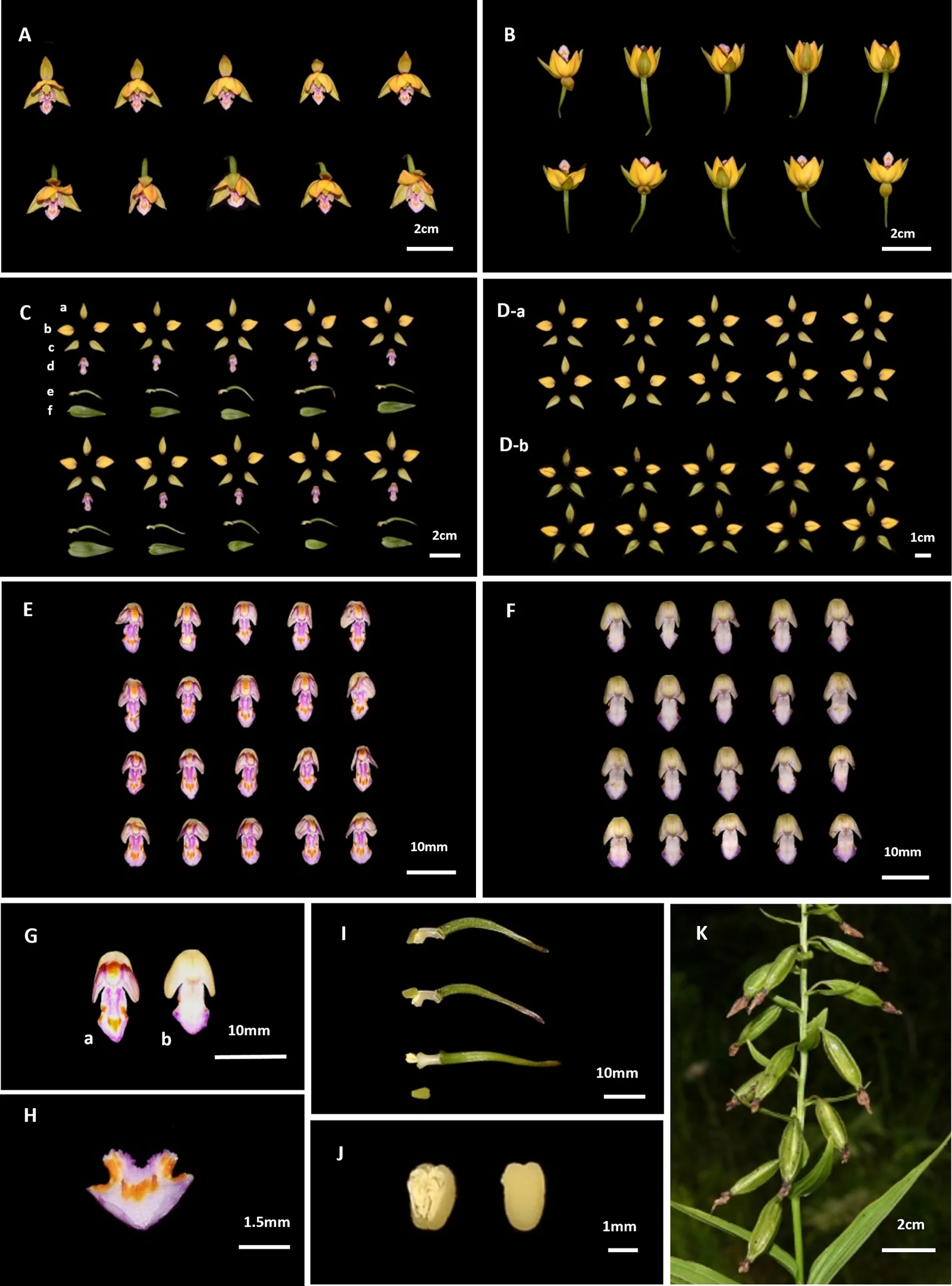
Floral morphology and dissected structures of *Epipactis thunbergii* from Korea. **A, B**. Various front and rear views of flowers; **C**. Structure of flower. a. dorsal sepal; b. lateral sepal; c. petal; d. lip; e. ovary and pedicel; f. floral bracts; **D**. a. various front views of sepals and petal; b. various rear views of sepals and petal; **E**. Various front views of lip; **F**. Various rear views of lip; **G**. Front and rear views of lip; **H**. Bottom view of callus; **I**. Ovary and pedicel; pollinia; and anther cap; **J**. Top and inner view of anther cap; **K**. Capsules. Photos by J.S. Lee.

Comparative morphological evidence confirms that Korean plants previously identified as *E. thunbergii* include *E. xanthophaea*, which is recognized here as a distinct species in Korea. These data demonstrate that a taxon previously misidentified as *E. thunbergii* corresponds to *E. xanthophaea* (Figs 1, 2, 3, 7–8). The diagnostic differences between *E. thunbergii* and *E. xanthophaea* are most clearly observed in the structure of the lip, particularly in the callus morphology (Figs 3, 8, Table 1). *Epipactis thunbergii* has a short mesochile and a hypochile bearing a pair of low ridges, whereas *E. xanthophaea* exhibits an elongated mesochile and a smooth hypochile lacking ridges. These differences provide the most reliable characters for distinguishing the two species. Both species belong to sect. *Arthrochilium* and are otherwise morphologically similar. *E. papillosa*, belonging to sect. *Epipactis*, differs clearly in lacking a mesochile and having a narrowly cordate to triangular hypochile. The varieties E. papillosa var. imkoeensis and E. papillosa var. sayekiana are treated as synonyms based on morphological comparison.

**Table 1.** Morphological comparisons of three *Epipactis* species in Korea.

**Characteristics**	***E. thunbergii***	***E. xanthophaea***	***E. papillosa***
**Plant height**	30–70 cm	40–90 cm	30–80 cm
**Leaf shape**	lanceolate to ovate-lanceolate	elliptic to ovate-elliptic	ovate to broadly ovate
**Inflorescence**	Glabrous	papillose	papillose-pubescent
**Flower color**	dark to light greenish-yellow	yellowish-brown, yellowish-red to red	pale green to green
**Lip**	3-lobed	3-lobed	not 3-lobed
**Hypochile**	cymbiform, with ridges	cymbiform, without ridges	subglobose-saccate
**Mesochile**	short, inconspicuous	elongated, distinct	absent
**Epichile**	obovate, with low keels	ovate-orbicular, without keels	triangular

The distribution patterns of the three species were re-evaluated using herbarium specimens and literature data. The three species also differ in habitat preference. *E. thunbergii* typically occurs in moist meadows, forest margins, and coastal habitats, whereas *E. xanthophaea* is most frequently associated with wetlands, marshy areas, and stream margins. In contrast, *E. papillosa* occurs mainly in shaded deciduous or mixed forests. *Epipactis thunbergii* occurs in Korea, Japan, and eastern China (Chen et al. 2009; POWO 2025; GBIF 2025), whereas *E. xanthophaea* is distributed in Korea, China, and the Russian Far East. Its occurrence in Korea bridges the previously known distribution between northeastern China and the Russian Far East. *Epipactis papillosa* is also confirmed as occurring in Russia based on previous studies (Komarov 1935; Efimov 2004, 2020; Fateryga et al. 2018b). A review of historical literature and herbarium specimens, including materials preserved in LE (Komarov 1901; Schlechter 1922; Tang and Wang 1951; Efimov 2004, 2018; Fateryga and Fateryga 2018a), supports treating Epipactis gigantea var. manshurica as conspecific with *E. xanthophaea* rather than *E. thunbergii*. This interpretation is supported primarily by concordant lip morphology and further supported by differences in plant habit, leaf shape, and leaf attachment. Accordingly, *E. xanthophaea* is retained as the correct name under the principle of priority (Turland et al. 2018).

*Epipactis xanthophaea* is newly recognized in Korea. Three species of *Epipactis* (*E. thunbergii*, *E. xanthophaea*, and *E. papillosa*) are confirmed on the Korean Peninsula. Their distinctions are supported primarily by lip morphology and supplemented by differences in distribution and habitat preference. This finding also clarifies the distributional continuity of *E. xanthophaea* across northeastern China, the Korean Peninsula, and the Russian Far East.

### Key to the species of *Epipactis* in Korea

**Table:** 

1	Lip 3-lobed, hypochile cymbiform; rhizome creeping, with elongated (> 2 cm) internodes (sect. *Arthrochilium*)	**2**
–	Lip not 3-lobed, hypochile subglobose-saccate; rhizome abbreviated, with short (< 2 cm) internodes (sect. *Epipactis* s.str.)	***E. papillosa***
2	Lip 10–11 mm, with a short mesochile, 1–2 mm; epichile with a pair of low keels	***E. thunbergii***
–	Lip 14–15 mm, with an elongated mesochile, 5–6 mm; epichile without keels	***E. xanthophaea***

### Taxonomic treatment

#### Epipactis thunbergii

Taxon classificationPlantaeAsparagalesOrchidaceae

A. Gray, Narr. Exp. China Japan 2: 319 (1856).—TYPE: JAPAN. Shizuoka, Shimoda, s.d., S.W. Williams & J. Morrow (holotype: GH [GH00052493]!)

2C67E362-EEBF-52CB-9E77-1AF1A0862681

Figs 2, 3, 4, 5

≡ Limodorum thunbergii (A. Gray) Kuntze, Revis. Gen. Pl. 2: 672 (1891).
≡ Helleborine thunbergii (A. Gray) Druce, Bull. Torrey Bot. Club 36: 547 (1909).
≡ Amesia thunbergii (A. Gray) A. Nelson & J.F. Macbr., Bot. Gaz. 56: 473 (1913).
≡ Epipactis gigantea var. thunbergii (A. Gray) M. Hiroe, Orchid Flowers 1: 63 (1971).
≡ Arthrochilium thunbergii (A. Gray) Szlach., Orchidee (Hamburg) 54: 588 (2003).
= Helleborine chinensis Soó, Ann. Hist.-Nat. Mus. Natl. Hung. 26: 380 (1929).
= Epipactis thunbergii f. subconformis Sakata, J. Jap. Bot. 35: 224 (1960).
= Epipactis thunbergii f. flava Ohwi, Fl. Jap., ed. rev.: 1438 (1965).

##### Korean name.

Dalg-ui-nancho (닭의난초).

**Figure 4.**
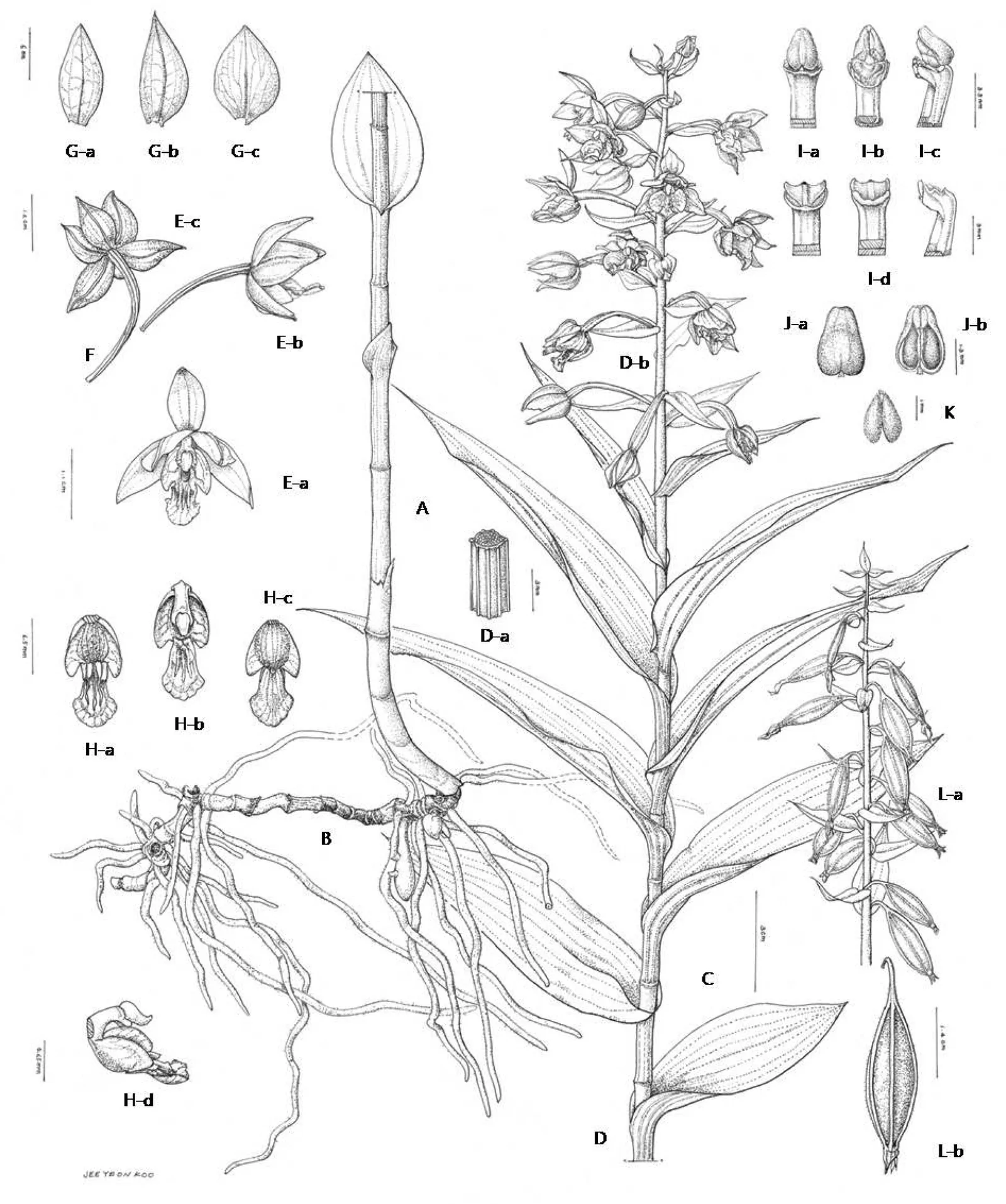
*Epipactis thunbergii* from Korea. **A**. Plant; **B**. Rhizome; **C**. Leaves; **D**. Inflorescence and flowers. a. cross-section of stem; b. floral bracts; **E**. Flower. a. front view; b. side view; c. rear view; **F**. Ovary and pedicel; **G**. Structure of flower. a. dorsal sepal; b. lateral sepal; c. petal; **H**. Lip. a. front view; b. top view of anther cap, column, and lip; c. rear view; **I**. Various views of column. a. top view of anther cap on column; b. front view; c. side view with anther cap open; d. various views without anther cap; **J**. Anther cap. a. top view; b. inner view with pollinia; **K**. Pollinia; **L**. Fruiting plant and capsules. a. fruiting inflorescence; b. capsule. Drawn by J.Y. Koo from flowering material: J.S. Lee LJS24-373 (KB), and fruiting material: J.S. Lee LJS24-377 (KB).

**Figure 5.**
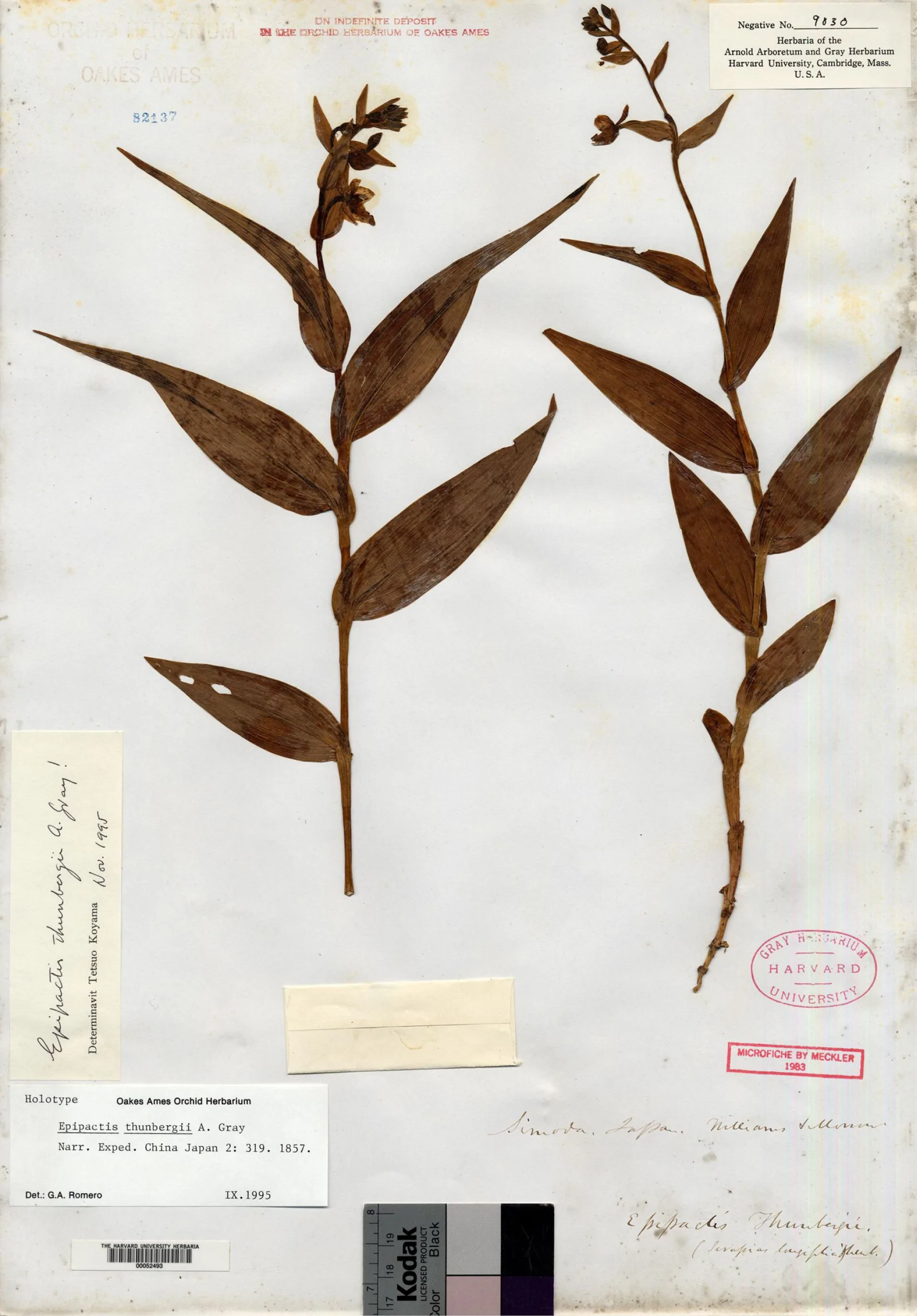
Holotype specimen of *Epipactis thunbergii* A. Gray (GH00052493).

##### Common name.

Thunberg’s helleborine.

##### Description.

Terrestrial herbs. Plants 30–70 cm tall. Rhizome elongate, creeping, with internodes, 2–4 (7) cm; roots fleshy, long, horizontal, with many roots arising from the internodes. Stem erect, glabrous and green, purplish only at base, with 3–5 basal sheaths. Leaves 6–9, uniformly arranged, lanceolate to ovate-lanceolate, 5–13 × 1.5–3.5 cm, glabrous, base shortly petiolate and amplexicaul, apex acuminate to long acuminate. Inflorescence terminal, racemose, glabrous; rachis 5–15 cm, laxly 3–15 (20)-flowered; floral bracts 2–3.3 × 0.5–1.2 cm, horizontal or ascending, lower floral bracts foliaceous, exceeding flowers, much longer than upper ones, ovate-elliptic, apex acute. Flowers spreading, resupinate, fully opening, dark to light greenish-yellow; lip white-purple to yellow-purple, with dark purple to light purple venation on side lobes and orange spots on disk; ovary and pedicel 15–22 mm long, clavate, ribbed, curved, glabrous or slightly pubescent, green to brownish-yellow. Sepals dark to light greenish-yellow; dorsal sepal elliptic, 10–11 × 4–4.5 mm, apex acute; lateral sepals ovate-lanceolate, oblique, 10–11 × 4–5 mm, apex acuminate. Petals broadly ovate, slightly oblique, 9–10 × 5–6 mm, apex acute. Lip 3-lobed, 10–11 × 8–9 mm, with two deeply split lateral lobes spreading outward, and a short mesochile connecting the hypochile and epichile; hypochile cymbiform concave, with lateral lobes, 4–5 × 8–9 mm, inside with purplish striations; lateral lobes erect 3.5–4 × 2 mm; mesochile sometimes inconspicuous, oblong, 1–2 mm; epichile obovate, when flattened, panduriform, 4–5 × 3–3.5 mm, margin slightly undulate, with a pair of low keels and a purplish blotch between them. Column 6–7 mm, including anther, glabrous, terete, relatively thick, and expanded into porrect wings. Rostellum ovate, bearing a lens-shaped viscidium positioned between two globose stigma edges. Anther narrowly ovate, pyriform, 2.5–3 mm, and papillate, pale green; pollinia 2, mealy. Capsule pendulous, ellipsoid, 18–28 × 8–10 mm. Chromosome number: 2n = 40 (Chen et al. 2009; Inoue 2016).

##### Flowering and fruiting.

Flowering June–July; fruiting July–August.

##### Habitat.

Sunny to semi-shaded humid meadows, forest swamp edges, moist valley edges, and moist rocky areas along the coast.

##### Distribution.

**Figure 6.**
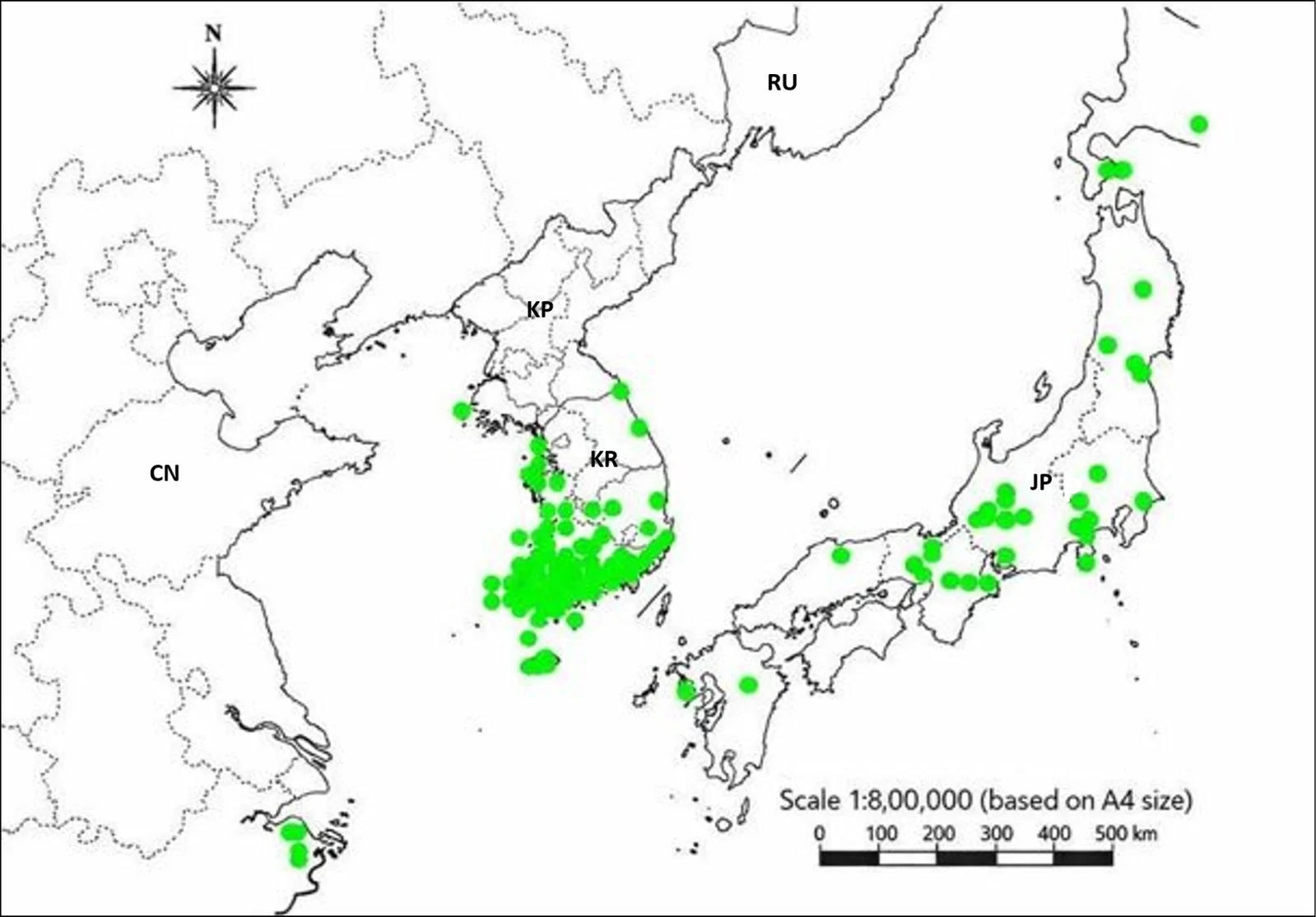
Distribution of *Epipactis thunbergii* in Korea, China, and Japan. Abbreviations: CN, China; KP, Democratic People’s Republic of Korea; KR, Republic of Korea; JP, Japan.

China (Zhejiang), Japan, Korea (Chen et al. 2009; GBIF 2025) (Fig. 6).

##### Taxonomic note.

*Epipactis thunbergii* is characterized by a short and sometimes inconspicuous mesochile and a cymbiform hypochile with two deeply split lateral lobes. The epichile bears a pair of low keels. In contrast, *E. xanthophaea* has an elongated mesochile (5–6 mm), a smooth hypochile lacking ridges, and an epichile without keels. These differences in lip structure provide reliable diagnostic characters distinguishing the two species (Table 1).

#### Epipactis xanthophaea

Taxon classificationPlantaeAsparagalesOrchidaceae

Schltr., Repert. Spec. Nov. Regni Veg. Beih. 12: 341 (1922).—TYPE: CHINA. Tschili: Jehol, feuchte Plätze am Passe Ba buo tse ling, nördlich Ma lan yün, im Bannwalde d. östlichen Kaisergräber, 4 July 1917, Limpricht H.W. 2915 (lectotype, designated here: WU [WU0061592]!)

F18B1EFC-D988-51E1-85EF-0599936B9010

Figs 7, 8, 9, 10

≡ Amesia xanthophaea (Schltr.) Hu, Rhodora 27: 106 (1925).
≡ Helleborine xanthophaea (Schltr.) Soó, *Ann. Hist.-Nat. Mus. Natl. Hung*. 26: 381 (1929).
≡ Arthrochilium xanthophaeum (Schltr.) Szlach., Orchidee (Hamburg) 54: 588 (2003).
= Epipactis gigantea var. manshurica Maxim. ex Kom., *Trudy Imp. S.-Peterburgsk. Bot. Sada* 20: 524 (1901).
= Epipactis thunbergii var. manshurica (Maxim. ex Kom.) Tang & F.T. Wang, *Acta Phytotax. Sin*. 1: 67 (1951).

##### Korean name.

Jang-dalg-ui-nancho (장닭의난초).

**Figure 7.**
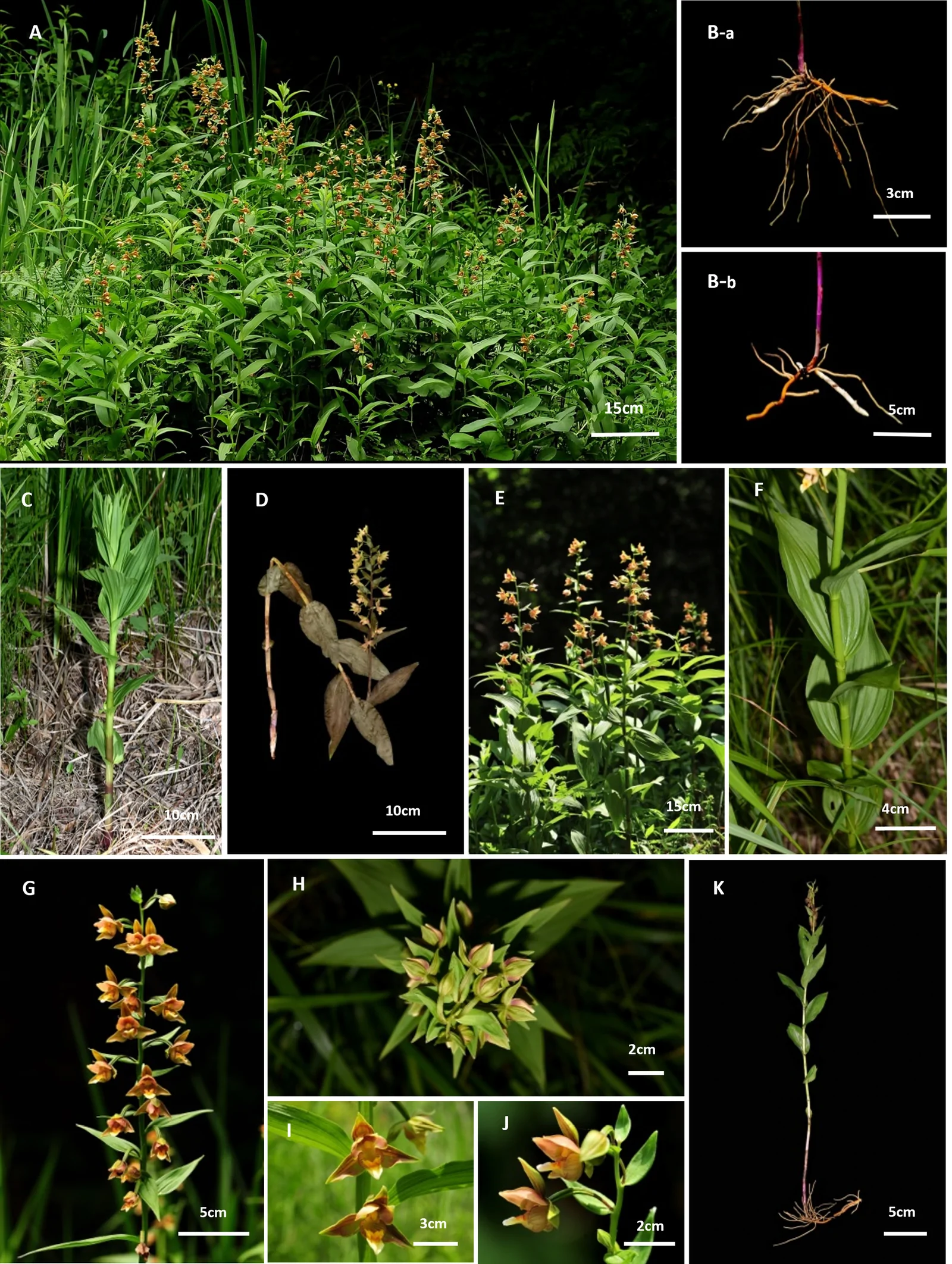
Photographs of *Epipactis xanthophaea* from Korea. **A**. Habit; **B**. a-b. Various views of rhizome; **C**. Young plants; **D**. Flowering plant; **E**. Plants in flower; **F**. Leaves; **G**. Inflorescence; **H**. Top view of inflorescence; **I**. Front view of flowers; **J**. Side view of flowers; **K**. Fruiting plant. Photos by J.S. Lee.

**Figure 8.**
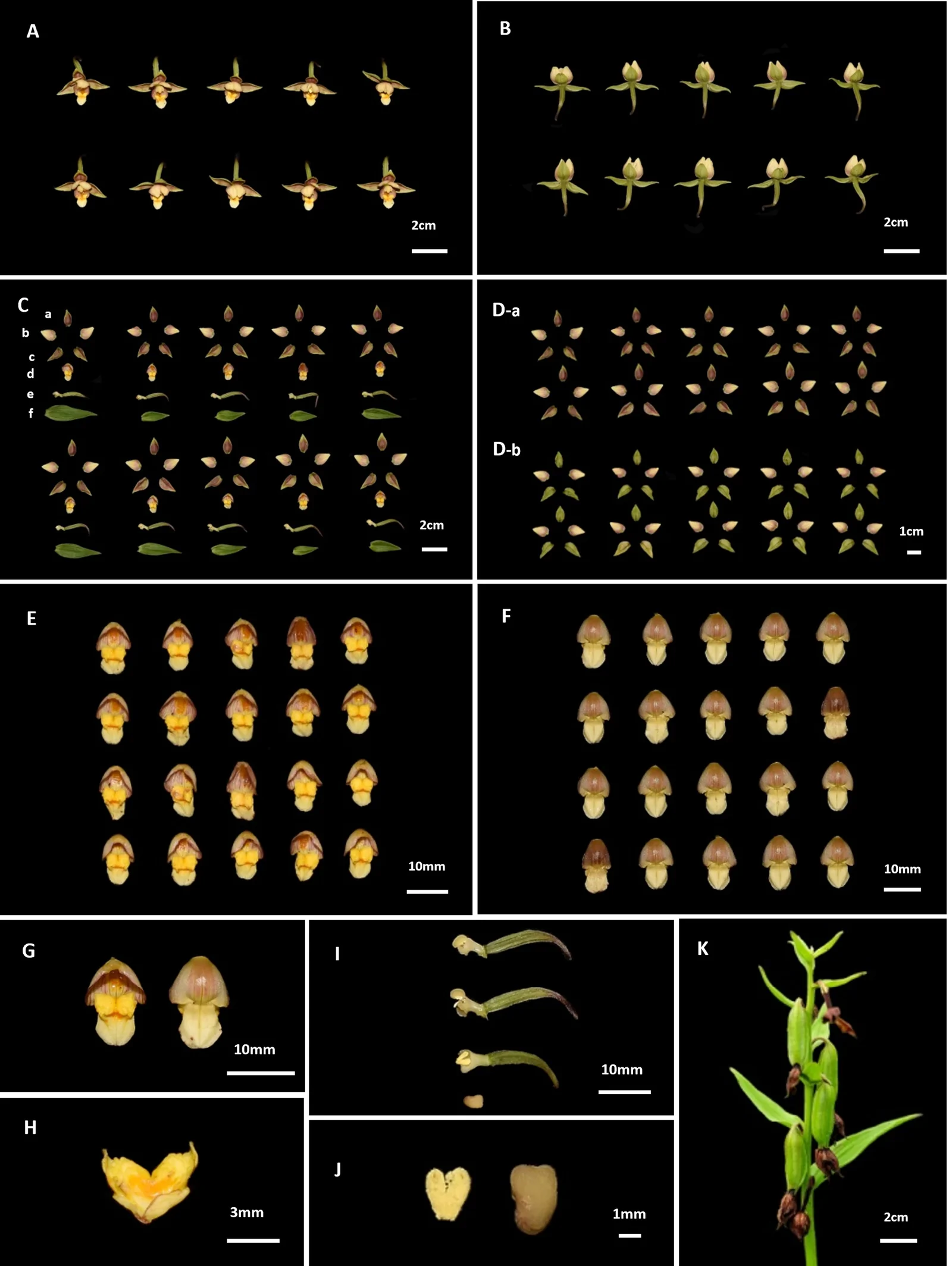
Floral morphology and dissected structures of *Epipactis xanthophaea* from Korea. **A, B**. Various front and rear views of flowers; **C**. Structure of flower. a. dorsal sepal; b. lateral sepal; c. petal; d. lip; e. ovary and pedicel; f. floral bracts; **D**. a. various front views of sepals and petal; b. various rear views of sepals and petal; **E**. Various front views of lip; **F**. Various rear views of lip; **G**. Front and rear views of lip; **H**. Bottom view of callus; **I**. Ovary and pedicel; pollinia; and anther cap; **J**. Pollinia and anther cap; **K**. Capsules. Photos by J.S. Lee.

**Figure 9.**
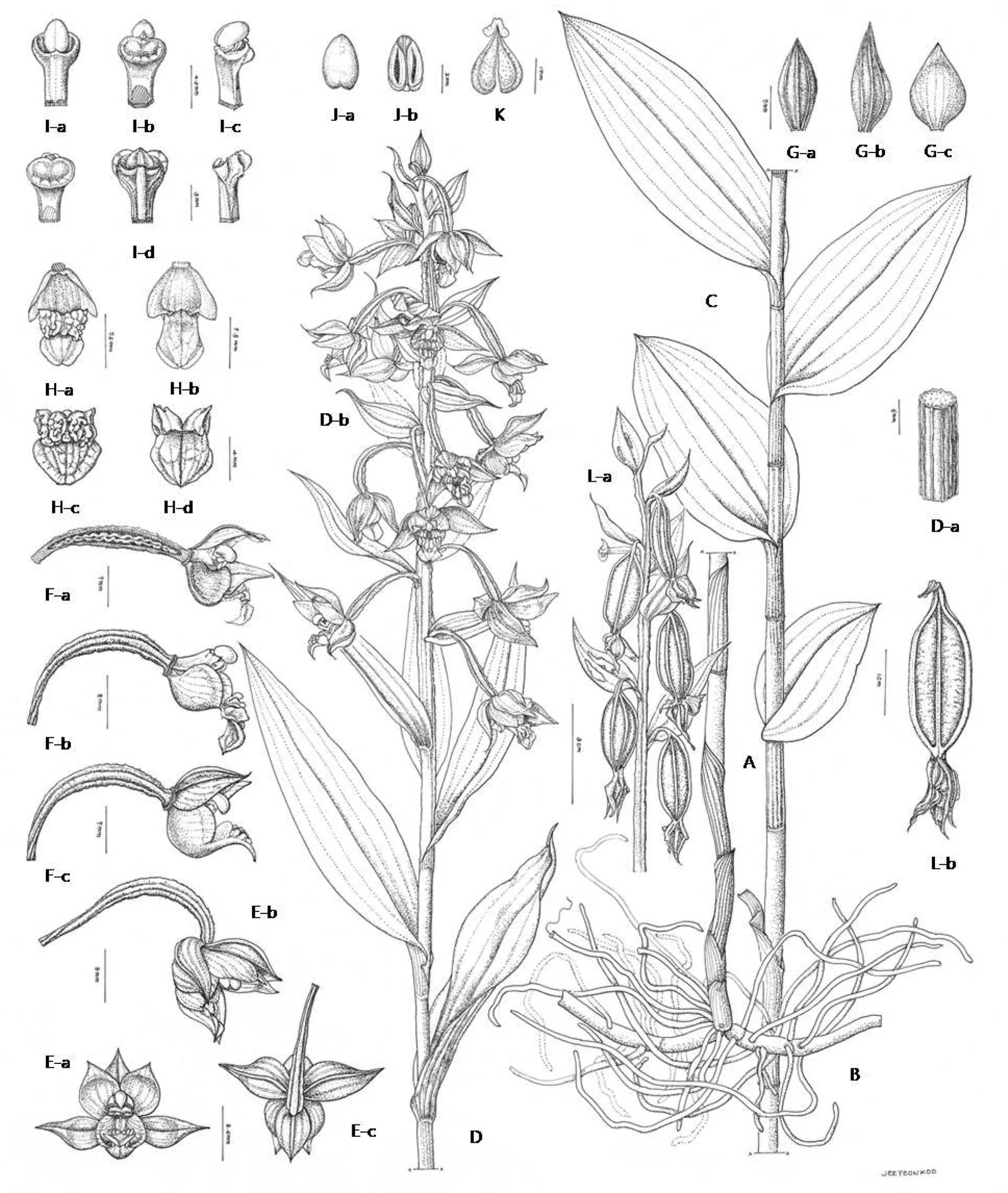
*Epipactis xanthophaea* from Korea. **A**. Plant; **B**. Rhizome; **C**. Leaves; **D**. Inflorescence and flowers. a. cross-section of stem; b. floral bracts; **E**. Flower. a. front view; b. side view; c. rear view; **F**. Various side views of the ovary and pedicel, lip, and column. a. cross-section of the ovary and pedicel; b. side views of the lip and column; c. side views of the petals and lip; **G**. Structure of the flower. a. dorsal sepal; b. lateral sepal; c. petal; **H**. Lip. a. front view; b. rear view; c. front view of the mesochile and epichile; d. bottom view of the mesochile and epichile; **I**. Various views of column. a. top view of anther cap on column; b. front view; c. side view with anther cap open; d. various views without anther cap; **J**. Anther cap. a. top view; b. inner view with pollinia; **K**. Pollinia; **L**. Fruiting plant and capsules. a. fruiting inflorescence; b. capsule. Drawn by J.Y. Koo from flowering material: J.S. Lee LJS24-391 (KB), and fruiting material: J.S. Lee LJS24-392 (KB).

**Figure 10.**
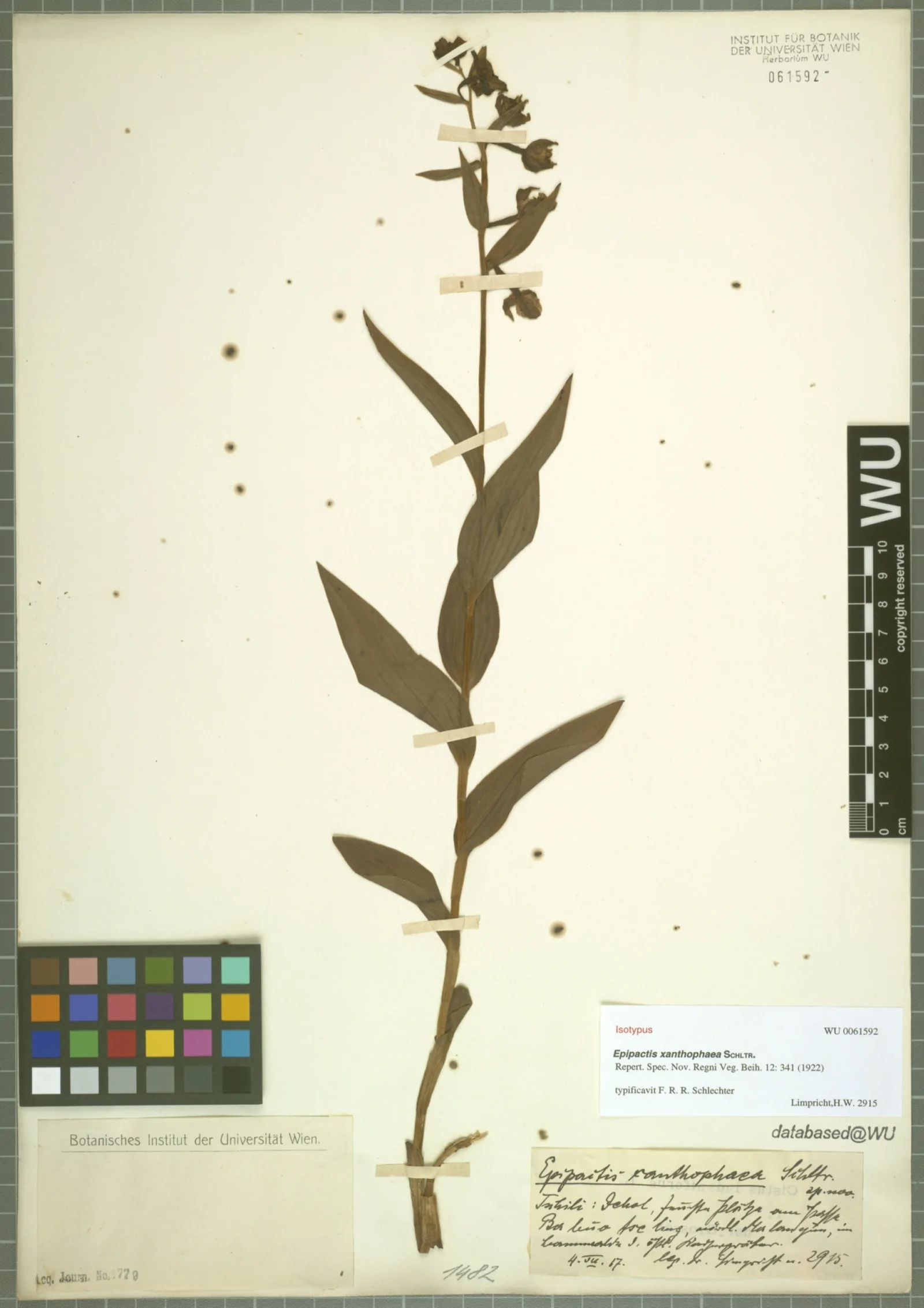
Lectotype specimen of *Epipactis xanthophaea* Schltr. (WU0061592).

##### Common name (proposed here).

Yellowish-red helleborine.

##### Description.

Terrestrial herbs. Plants 40–90 cm tall. Rhizome elongate, creeping, with internodes, 2–6 cm; roots slender, simple, long, and horizontal, with roots arising along the rhizome, including at the nodes and indistinct internodes. Stem erect, glabrous and green, purplish only at base, sparsely papillose towards the apex, with 4–7 (8) basal sheaths. Leaves 5–8, spreading, elliptic to ovate-elliptic, with rounded to broadly rounded bases, 6–16 × 4–6 (8) cm, glabrous, base amplexicaul, apex acute to sub-acuminate. Inflorescence terminal, racemose, glabrous; rachis 10–22 (28) cm, sub-laxly 5–20 (25)-flowered, subsecund; floral bracts 4–8 × 1–2 cm, horizontal or ascending, lower floral bracts foliaceous, exceeding flowers, much longer than upper ones, lanceolate or ovate-lanceolate, apex long acuminate. Flowers spreading, resupinate, fully opening, rather large, yellowish-brown, yellowish-red to red, or rarely pale red; lip yellowish-red, with yellow to pale yellow; ovary and pedicel 15–25 mm, clavate, ribbed, curved, finely papillose-puberulent, pale purplish green. Sepals yellowish-brown, yellowish-red to red; dorsal sepal elliptic, 14–15 × 5–6 mm, apex acute; lateral sepals ovate-lanceolate, oblique, 15–16 × 6–7 mm, apex acuminate. Petals broadly ovate, slightly oblique, 14–15 × 7–8 mm, apex acute. Lip 3-lobed, 13–15 × 8–12 mm, with slightly inward-facing, erect, ovate-suborbicular lateral lobes, and a broad mesochile connecting hypochile and epichile; hypochile cymbiform, concave, with lateral lobes, 6–8 × 11–12 mm, erect ovate-suborbicular, obtuse, with scattered verrucas and brownish-red striations along the median line at and above the base; mesochile 5–6 × 4–5 mm, with longitudinally protruding, obtuse lateral margins, dilated into a cordate outline, and a pair of subtriangular lamellae, 3.5 × 3 mm, enclosed by a thin membrane, yellow; epichile ovate-orbicular, 5–6 × 4–5 mm, margin smooth, without keels, pale yellow. Column 5–6 mm, including anther, glabrous, terete, thick, and expanded into porrect wings. Rostellum ovate, bearing a lens-shaped viscidium positioned between two large, trapezoidal-rhombic stigma edges. Anther long ovoid, 3–3.5 mm, pale brownish-yellow; pollinia 2, mealy. Capsule pendulous, oblong-ellipsoid, 25–32 × 10–20 mm.

##### Flowering and fruiting.

Flowering June–July; fruiting July–August.

##### Habitat.

Sunny and moist areas with muddy wetland soil, such as the edges of wetlands, within wetlands, and the edges of waterways covered with trees or grass. The rhizomes of the plant are submerged in a mixture of silt and water deposited in the wetland.

##### Distribution.

**Figure 11.**
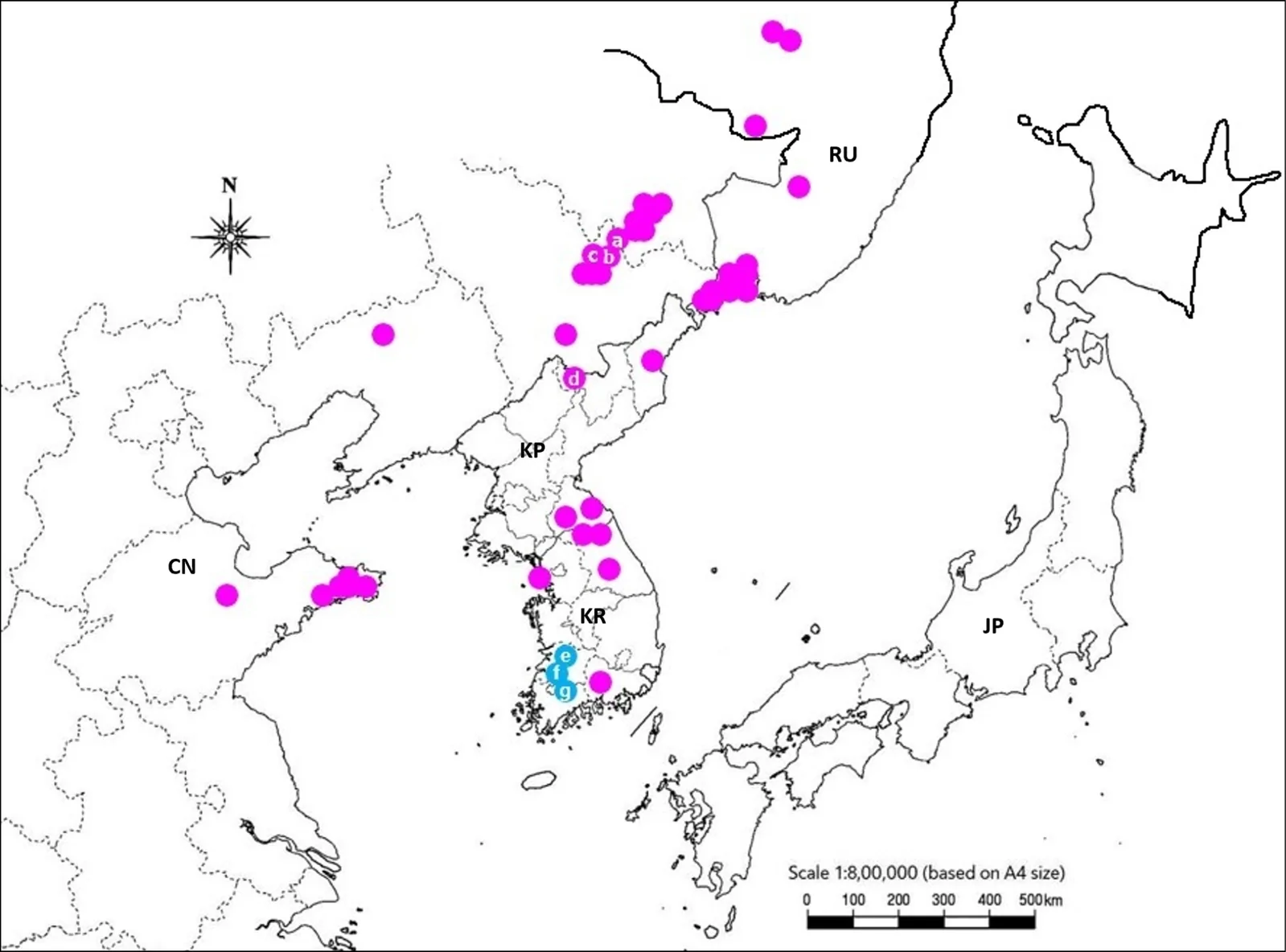
Distribution of *Epipactis xanthophaea* in Korea, China, and Russia; a-d. Collection sites documented by Komarov in 1896 and 1897; e-g. historical localities where the species was not relocated during recent field surveys. Abbreviations: CN, China; KP, Democratic People’s Republic of Korea; KR, Republic of Korea; RU, Russian Federation.

China (Hebei, Heilongjiang, Jilin, Liaoning, Shandong), Russia (Far East), Korea (Chen et al. 2009; POWO 2025) (Fig. 11).

##### Taxonomic note.

*Epipactis xanthophaea* was described by Schlechter (1922) based on material collected in Jehol, China. The species has long been poorly understood and frequently misinterpreted as *E. thunbergii* in regional floras. A review of the literature and herbarium specimens indicates that Epipactis gigantea var. manshurica Maxim. ex Kom. is conspecific with *E. xanthophaea* rather than *E. thunbergii*. Although originally compared with *E. gigantea*, this taxon differs clearly from that species in plant size, floral morphology, and lip structure (Brown and Argus 2002). Russian plants previously referred to *Epipactis thunbergii* are better interpreted as *E. xanthophaea* based on concordant morphology, supported by literature, herbarium specimens, and photographic evidence (Regel 1861: 145; Komarov 1901, 1935; Efimov 2004, 2018; Kozhevnikov et al. 2015, 2019; Fateryga and Fateryga 2018a). *Epipactis xanthophaea* was previously considered to be restricted to northeastern China (Chen et al. 2009; POWO 2025; Plant Photo Bank of China 2025; iPlant.cn 2025; Chinese Virtual Herbarium 2025). However, it is now known to occur in the Russian Far East and Korea, extending from the southern slopes of the Baekdudaegan to central and northeastern regions. Specimens collected by Komarov from northeastern China and northern Korea and preserved at LE provide evidence of a continuous distribution across this region (Kropotkin 1898; Komarov 1901, 1935; Fig. 11a–d; LE01042583, LE01042584, LE01012227, LE01042585).

In Korea, its occurrence is confirmed in four northern regions based on herbarium specimens and in eight southern regions based on specimens and field observations. However, its presence in three regions remains unconfirmed (Fig. 11e–g): one is supported only by photographs (Fig. 11f), another is based solely on historical records (Fig. 11g; TI00014572), and the third is represented by a single specimen without recent confirmation (Fig. 11e; KH1420121). Accordingly, the confirmed distribution in Korea includes Jagang-do (Huchang-gun), Hamgyeongbuk-do, Gangwon-do, Gyeongsangnam-do, and Incheon (Fig. 11).

##### Lectotype designation of *Epipactis xanthophaea* Schltr.

No holotype was designated in the protologue of *Epipactis xanthophaea* Schltr., and the original material is presumed to have been deposited in the Berlin Herbarium (B), where it was likely destroyed during the air raids of 1943 (Ames 1944). In accordance with Articles 9.3, 9.11, and 9.12 of the International Code of Nomenclature for algae, fungi, and plants (Turland et al. 2018), the specimen Limpricht 2915 (WU0061592), collected by the original collector and currently preserved at WU, is designated here as the lectotype.

#### Epipactis papillosa

Taxon classificationPlantaeAsparagalesOrchidaceae

Franch. & Sav., Enum. Pl. Jap. 2: 519 (1878).—TYPE: JAPAN. Yéso [Hokkaido], 1874, K.U. Kramer s.n. (type: P [P00301867]!)

96F758C9-E314-58D5-9FB7-F2E8AE13DF29

Figs 12, 13, 14, 15

≡ Limodorum papillosum (Franch. & Sav.) Kuntze, Revis. Gen. Pl. 2: 672 (1891).
≡ Epipactis latifolia var. papillosa (Franch. & Sav.) Maxim. ex Kom., *Fl. Manshur*. 1: 523 (1901).
≡ Helleborine papillosa (Franch. & Sav.) Druce, Vierteljahrsschr. Naturf. Ges. Zürich 53: 589 (1908).
≡ Amesia papillosa (Franch. & Sav.) A. Nelson & J.F. Macbr., Bot. Gaz. 56: 472 (1913).
≡ Epipactis helleborine var. papillosa (Franch. & Sav.) T. Hashim., Proc. World Orchid Conf. 12: 120 (1987).
≡ Epipactis helleborine subsp. papillosa (Franch. & Sav.) Fateryga, Turczaninowia 21(4): 24 (2018).
**=**Epipactis sayekiana Makino, J. Jap. Bot. 2: 22 (1918).
**=**Serapias sayekiana (Makino) Makino, J. Jap. Bot. 6: 8 (1929).
**=**Epipactis papillosa var. sayekiana (Makino) T. Koyama & Asai, J. Jap. Bot. 33: 226 (1958).
**=**Epipactis helleborine var. sayekiana (Makino) T. Hashim., Proc. World Orchid Conf. 12: 121 (1987).
**=**Epipactis papillosa var. imkoeensis Y.N. Lee & K.S. Lee, Bull. Korea Pl. Res. 2: 38 (2002).

##### Korean name.

Cheong-dalg-ui-nancho (청닭의난초).

**Figure 12.**
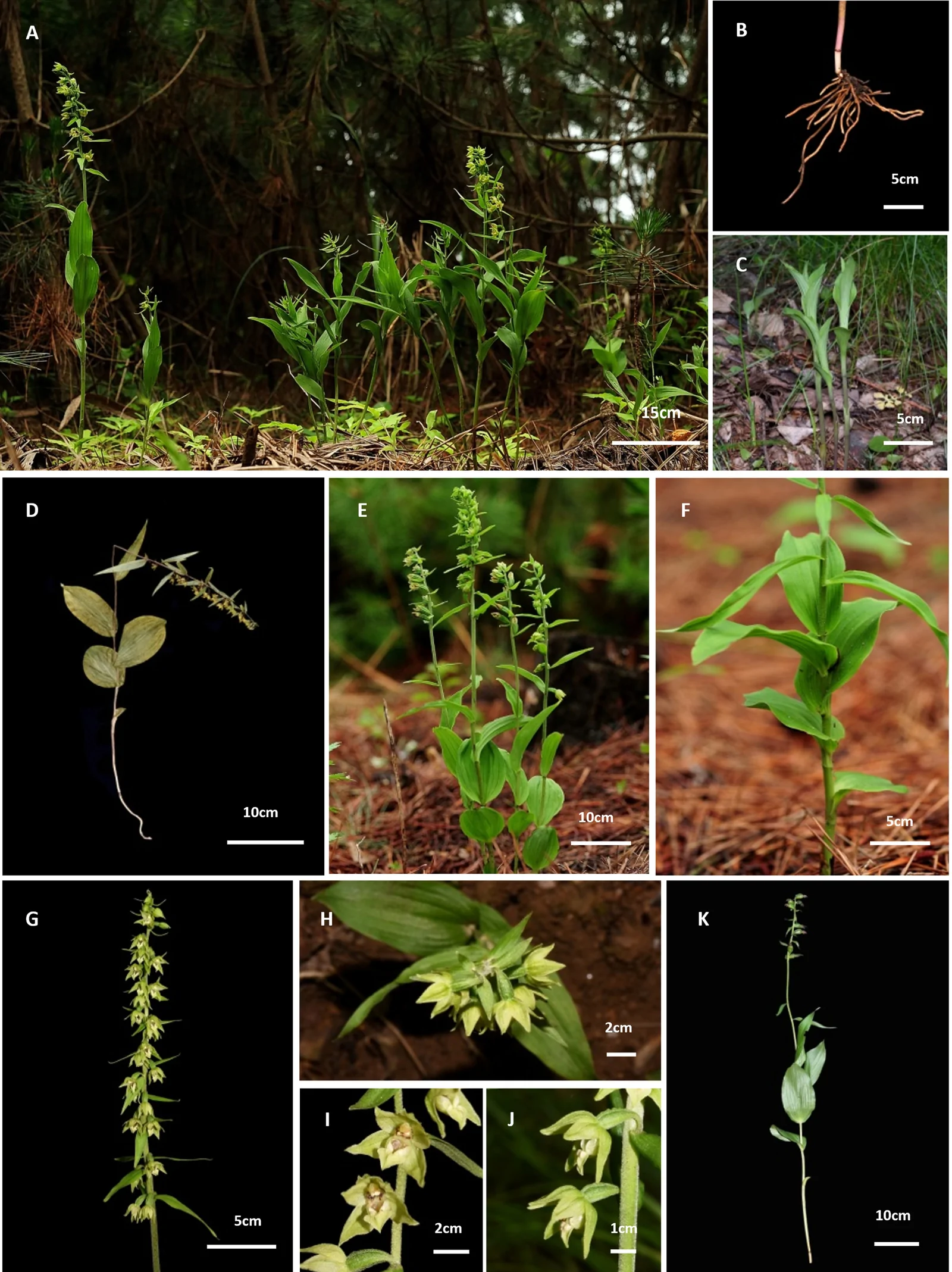
Photographs of *Epipactis papillosa* from Korea. **A**. Habit; **B**. Views of rhizome; **C**. Young plants; **D**. Flowering plant; **E**. Plants in flower; **F**. Leaves; **G**. Inflorescence; **H**. Top view of inflorescence; **I**. Front view of flowers; **J**. Side view of flowers; **K**. Fruiting plant. Photos by J.S. Lee.

**Figure 13.**
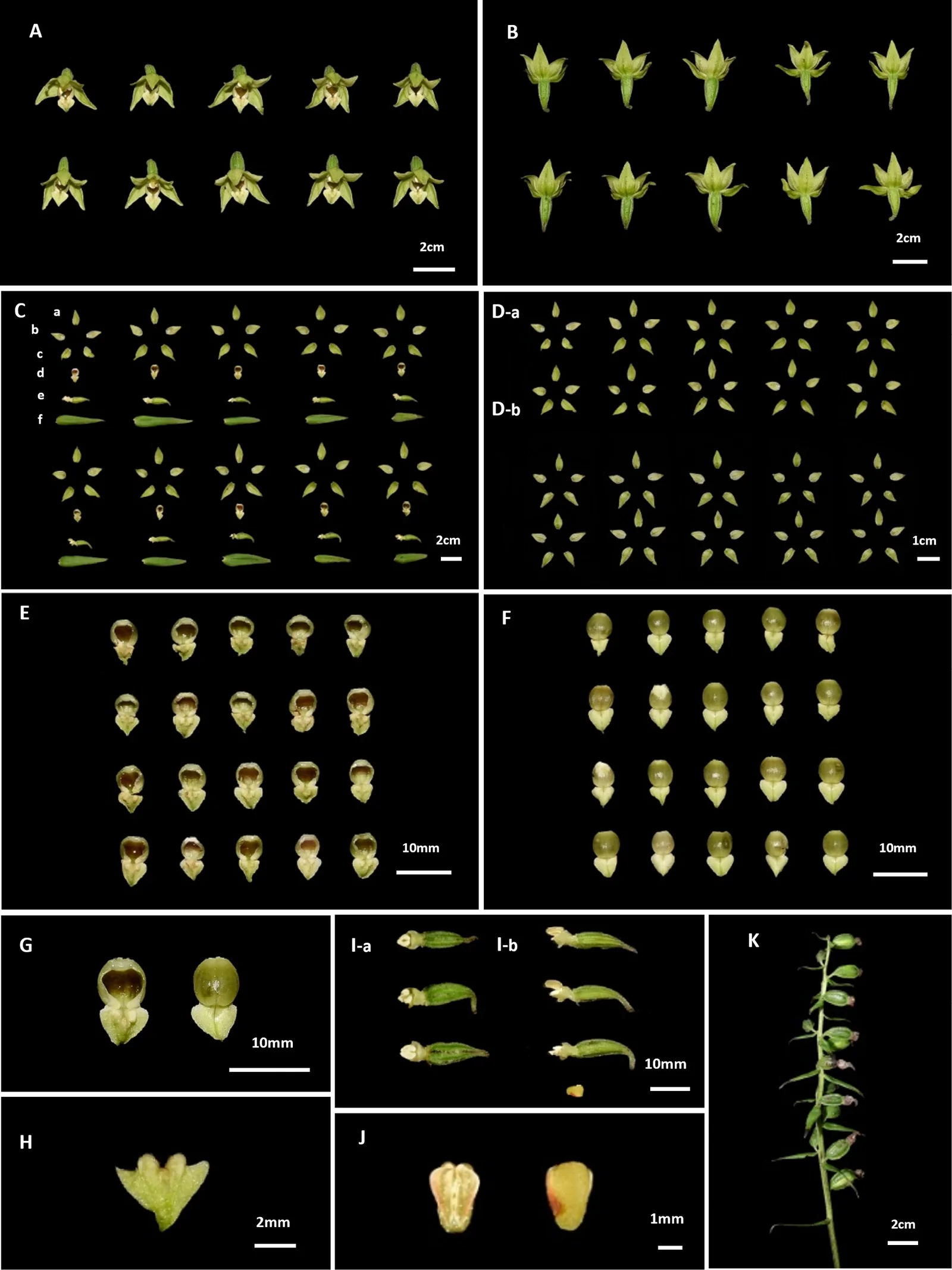
Floral morphology and dissected structures of *Epipactis papillosa* from Korea. **A, B**. Various front and rear views of flowers; **C**. Structure of flower. a. dorsal sepal; b. lateral sepal; c. petal; d. lip; e. ovary and pedicel; f. floral bracts; **D**. a. various front views of sepals and petal; b. various rear views of sepals and petal; **E**. Various front views of lip; **F**. Various rear views of lip; **G**. Front and rear views of lip; **H**. Bottom view of callus; **I**. a-b. Ovary and pedicel; pollinia; and anther cap; **J**. Top view of pollinia and anther cap; **K**. Capsules. Photos by J.S. Lee.

**Figure 14.**
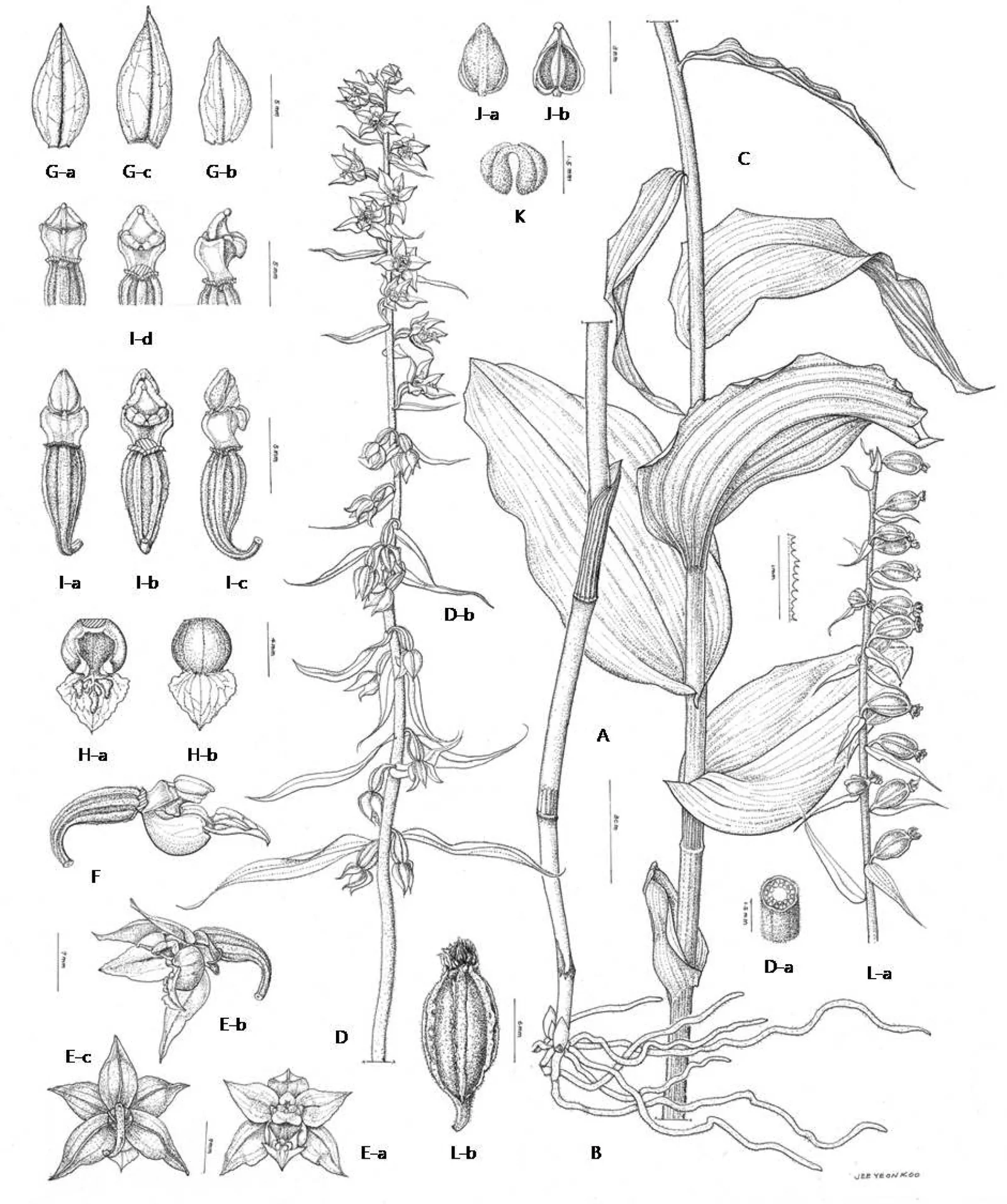
*Epipactis papillosa* from Korea. **A**. Plant; **B**. Rhizome; **C**. Leaves; **D**. Inflorescence and flowers. a. cross-section of stem; b. floral bracts; **E**. Flower. a. front view; b. side view; c. rear view; **F**. Side views of the ovary and pedicel, column and lip; **G**. Structure of flower. a. dorsal sepal; b. lateral sepal; c. petal; **H**. Lip. a. front view; b. rear view; **I**. Various views of column with ovary and pedicel. a. top view of anther cap on column; b. front view; c. side view with anther cap open; d. various views without anther cap; **J**. Anther cap. a. top view; b. inner view with pollinia; **K**. Pollinia; **L**. Fruiting plant and capsules. a. fruiting inflorescence; b. capsule. Drawn by J.Y. Koo from flowering material: J.S. Lee LJS24-384 (KB), and fruiting material: J.S. Lee LJS24-398 (KB).

**Figure 15.**
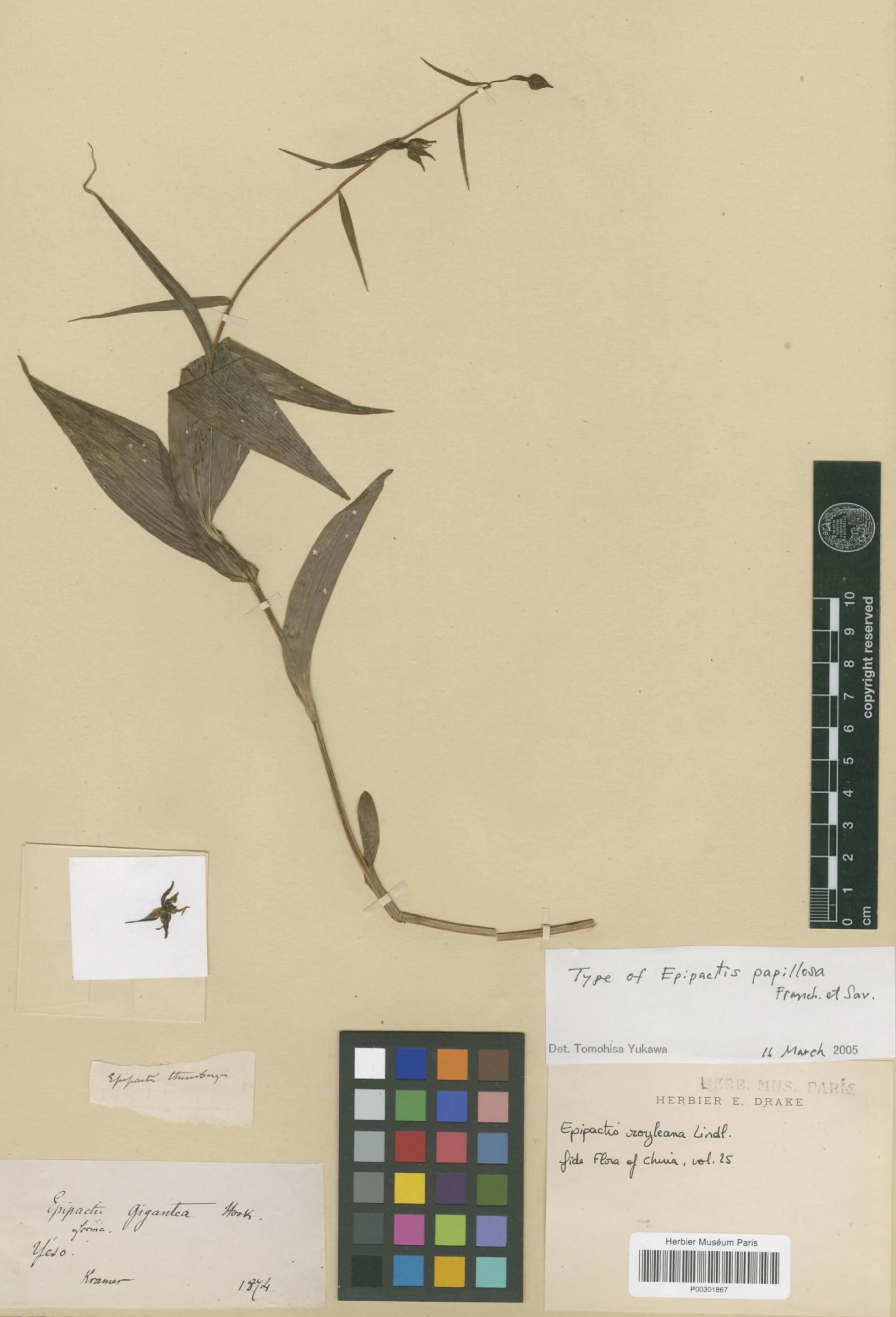
Type specimen of *Epipactis papillosa* Franch. & Sav. (P00301867).

##### Common name.

Green-flower helleborine.

##### Description.

Terrestrial herbs. Plants 30–80 cm tall. Rhizome with shortened internodes, 0.5–2 cm; roots fleshy, elongate, horizontal, becoming fibrous. Stem solitary, rarely 2–4 together, erect, uniformly brown and papillose-pubescent, with 3–4 basal sheaths; internodes elongated. Leaves 4–6, ovate to broadly ovate; upper cauline leaves ovate-lanceolate, 6–14 × 2–6 cm; lowermost leaf ovate, somewhat smaller; adaxially white papillose-puberulent along the veins and margins; apex acute to shortly acuminate. Inflorescence terminal, racemose, rather loosely many-flowered; rachis 10–20 cm, brown papillose-pubescent, laxly to subdensely 5–20 (30)-flowered; floral bracts 4–9 × 0.5–1.5 cm, horizontal or ascending; lower floral bracts foliaceous, exceeding the flowers, much longer than the upper ones, rough on the margin due to whitish papillae, linear to linear-lanceolate, with apex long, acute to acuminate. Flowers spreading horizontally or nodding, resupinate, rather pendent, not widely opening and likely self-pollinating, pale green to green; lip pale green; ovary and pedicel 12–18 mm, ribbed, green to dark green; ovary sparsely papillose; pedicels more or less pubescent. Sepals keeled dorsally, pale green to green; dorsal sepal elliptic, 8–9 × 3–4 mm, apex acute; lateral sepals ovate-lanceolate, oblique, 9–10 × 4–5 mm, apex acuminate. Petals keeled dorsally, broadly ovate, slightly oblique, 7–8 × 4–4.5 mm, apex acute. Lip not 3-lobed, entire, 7–8 × 4–4.5 mm, without mesochile; hypochile subglobose–saccate, deeply concave, ridges raised, without lateral lobes; epichile narrowly cordate or triangular, 3.5–4 × 3–3.5 mm, margin erose and slightly undulate, disc pendent, apex acute, with 2 globose calli at the base. Column 4–5 mm, short, terete, thick, and expanded into wings, with right-angled, papillate auricles at both apical corners. Rostellum ovate, depressed, large; viscidium globose; stigma porrect, semiglobose. Anther narrowly ovate, 2.5–3 mm, pale yellow; pollinia 2, mealy. Capsule horizontal or ascending, ovoid, 15–20 × 5–10 mm. Chromosome number: 2n = 38, 40 (Chen et al. 2009; Inoue 2016).

##### Flowering and fruiting.

Flowering June–July; fruiting July–August.

##### Habitat.

Shaded, moist forest edges; deciduous and mixed forests; primarily calcareous soils; tolerates moderately acidic soils.

##### Distribution.

**Figure 16.**
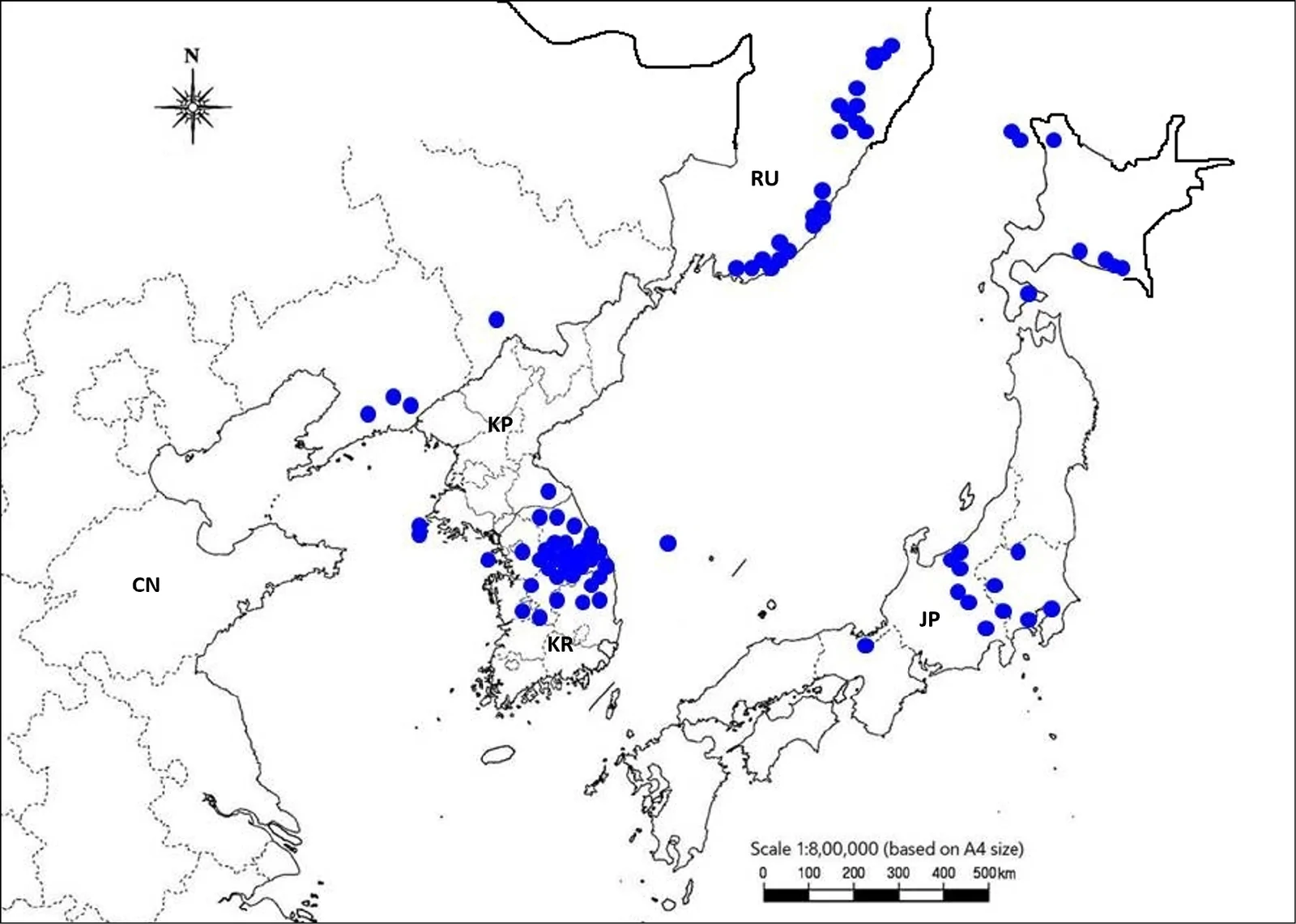
Distribution of *Epipactis papillosa* in Korea, China, Japan, and Russia. Abbreviations: CN, China; KP, Democratic People’s Republic of Korea; KR, Republic of Korea; JP, Japan; RU, Russian Federation.

China (Northeast), Japan, Korea, Russia (Kamchatka, Khabarovsk, Kuril Islands, Primorye, Sakhalin, Ussuri) (GBIF 2025; POWO 2025) (Fig. 16).

##### Taxonomic note.

*Epipactis papillosa* was described by Franchet and Savatier (1878) from Japan and belongs to sect. *Epipactis* s.str. It has often been compared with *E. helleborine*, which is widely distributed across Europe, North Africa, and Asia. In contrast, *E. papillosa* is restricted to East Asia, including Korea, northeastern China, the Russian Far East, and Japan (Chen et al. 2009; Efimov 2018, 2020; Fateryga et al. 2018b; GBIF 2025; POWO 2025). The taxonomic status of *E. papillosa* has been debated due to its morphological similarity to *E. helleborine* and has recently been treated as *E. helleborine* subsp. *papillosa* (Fateryga and Fateryga 2018a). However, *E. papillosa* differs in having brown, papillose-pubescent stems, fewer leaves and flowers, and a shorter lip (7–8 mm vs. 9–12 mm) (Brown and Argus 2002; Chen et al. 2009), supporting its recognition as a distinct species. *Epipactis sayekiana* and E. papillosa var. imkoeensis are here treated as synonyms of *E. papillosa*, as they do not show consistent morphological differentiation at the population level.

In Korea, sect. *Epipactis* is represented only by *E. papillosa*, which occurs in eastern coastal regions of Gyeongsang and Gangwon Provinces, inland areas of central and northern Korea, several western coastal islands, and northern regions (Fig. 16).

## Supplementary Material

XML Treatment for Epipactis thunbergii

XML Treatment for Epipactis xanthophaea

XML Treatment for Epipactis papillosa

## Additional information

### Artificial Intelligence (AI) use

The authors accept full responsibility for the content of the manuscript, including the disclosure of any use of AI.

No AI tools were used in the preparation of this manuscript.

### Author ORCIDs

J.-S. Lee https://orcid.org/0000-0003-2815-6464

J.-Y. Koo https://orcid.org/0009-0003-3204-0808

M.-H. Kim https://orcid.org/0009-0006-9203-7944

### Data availability

All of the data that support the findings of this study are available in the main text or Supplementary Information.

## Supplementary materials

Supplementary material 1 Examined specimens of three Epipactis species from Korea, China, Japan, and Russia Data type docx https://binary.pensoft.net/file/1751748This dataset is made available under the Open Database License (http://opendatacommons.org/licenses/odbl/1.0/). The Open Database License (ODbL) is a license agreement intended to allow users to freely share, modify, and use this Dataset while maintaining this same freedom for others, provided that the original source and author(s) are credited. Jung-Sim Lee, Jee-Yeon Koo, Min-Ha Kim
