# Supplementary material for: Taxonomic revision of Epipactis Zinn (Orchidaceae) on the Korean Peninsula
Source: PhytoKeys. 2026 Aug 21;278:205–31. doi: 10.3897/phytokeys.278.195337 (PMC13525408; doi:10.3897/phytokeys.278.195337)
Supplement: Supplementary file 1 [file phytokeys-278-205_article-195337__-s001.docx]

Supplementary material 1

Examined specimens of three Epipactis species from Korea, China, Japan, and Russia

**1. *Epipactis thunbergii*** A. Gray

**Korea.** **Gangwon-do:** Samcheok-si, Geundeok-myeon, 12 Jul 2000, *S.H. Park et al.* *s.n.* (2 sheets) (KH); Samcheok-si, Miro-myeon, 18 Jul 2000, *K.W. Park, Y.C. Choi, KNAH017506* (KH); same locality, 16 Jul 2000, *K.W. Park, Y.C. Choi KNAH017502* (KH); Samcheok-si, Wondeok-eup, Mt. Geombongsan, 27 Jul 2002, *E.S. Jeon* *s.n.* (KH); same locality, 28 Jun 2002, *E.S. Jeon* *s.n*. (2 sheets) (KH); Gosung-gun, Toseong-myeon, Cheonjin-ri, San 37, 23 m, 18 Aug 2014, *Y.C. Kim KYC2014-263* (KB). **Gyeongsangbuk-do:** Uljin-gun Buk-myeon, 24 Oct 2000, *G.Y. Chung, M.S. Park* *s.n.* (KB); Sangju-si, Mt. Sokrisan, Mt. Baekaksan, 28 Sep 2013, *B.U. Oh et al. Sangju-si-130928-035* (KH); Mungyeong-si, Dongro-myeon, Gongdeoksan, 451 m, 7 Jul 2007, *G.Y. Yeong* *s.n.* (3 sheets) (KB); Uljin-gun, Maehwa-myeon, Deoksin-ri, 17 Jul 2007, *G.Y. Chung ANH-en-070717-005* (KB). **Gyeongsangnam-do:** Geojedo Island, Aug 1912, *T. Mori 81* (TI); Prov. Keinan, Kariinji (Currently Haeinsa Temple), 21 Jul 1932, *Tukurhima, Nalcao s.n.* (KYO); Keinam, Mt. Chiisan, Jun 1935, *J. Ohwi 7131* (KYO); Mt. Tii., Yeongwonsa, 28 Jul 1937, *S. Okamota s.n.* (KYO); Geoje-si, Geoje-myeon, Oksan-ri, 127 m, 10 Sep 2010, *E.S. Jeon E.S.Jeon102476* (KH); same locality, 17 Jul 2007, *E.S. Jeon s.n.* (KH); Tongyeong-si, Bongpyeong-dong, Mireuksan, 151 m, 30 Jun 2014, *S.H. Yeo, C.S. Lee, H.J. Kim Leecs726* (KB); same locality, 30 Jun 2014, *S.H. Yeo, C.S. Lee, H.J. Kim Leecs727*, *Leecs728* (KB); Uiryeong-gun, Daei-myeon, Sinjeon-ri, 696 m, 2 Jul 2014, *H.J. Choi* *s.n.* (KB); Yangsan-si, Dongmyeon, Sasong-ri, Geumjeongsan, 714 m, 25 Jun 2016, *Y.S. Kim, H.J. Kim, J.U. Lee* *s.n.* (KH); Uiryeong-gun, Chilgok-myeon, Naejo-ri, Janggulsan, 730 m, 10 Sep 2017, *Y.H. Cho, S.S. Kim, S.H. Kim WR1915584* (KB); Gosung-gun, Ha-il-myeon, Hakrim-ri, Hyangnobong, San 117, 338 m, 6 Oct 2019, *Y.H. Cho, S.S. Kim s.n.* (KB); Hapcheon-gun, Gahoe-myeon, Jungcheon-ri, San 180, 865 m, 12 Jul 2020, *W.J. Lee, Y.S. Lee, Y.J. Jeong, S.J. Park GN200224* (KH); Ulsan, Mt. Dongdaesan Wetland, 31 Jul 2020, *J.S. Lee LJS200731-1* (2 sheets) (KB). **Busan:** Gijang-gun, Ilgwang-myeon, Samseong-ri, Ilgwangsan, 134 m, 21 Oct 2012, *J.O. Hyun* *s.n.* (KB); Gijang-gun, Gijang-eup, Jukseongri, 19 m, 12 Aug 2012, *G.Y. Byeon s.n.* (KB). **Jeju-do:** Quelpart, Oct 1906, *U. Faurie 1447* (KYO); Herbidis Hallaisan, 1,200 m, Jul 1907, *U. Faurie 2062* (P); Quelpart in sylvis Hallasan, 1,000 m, 13 Aug 1908, *L. Taquet 1648* (K); same locality, 2,000 m, 13 Aug 1908, *L. Taquet 1649* (K); Quelpart. Hannasan, 19 Aug 1930, *S.* *Kitamura s.n.* (KYO); Mt. Hallasan, 22 Jun 2004, *N.S. Lee, S.H. Yeo, C.S. Lee D-98, D-96* (2 sheets) (KB); Seogwipo-si, Hawon-dong, Mt. Hallasan Bolleoreum, 1,230 m, 18 Sep 2009, *s.n. 27744* (KB); Jeju City, Aewol-eup, Yusuam-ri, Geombaengdwi, 991 m, 23 Jul 2009, *J.C. Yang, C.S. Jang, J.W. Jang K11721* (KH); Seogwipo-si, Sanghyo-dong, Seondol, 403 m, 24 Jun 2010, *J. Kim 29865* (KB); Haean-dong, Mt. Hallasan, 26 Sep 2011, *M.O. Moon, J.E. Kim 2497* (KB); Bukjeju-gun, Chuja-myeon, Mukri, Dongdaesan, 30 m, 10 Jul 2012, *M.S. Lee, G.A. Jeong* *s.n.* (KB); Chujamyeon, Chujado, Hachujado, 20 m, 20 Jun 2012, *D.S. Kim, S.Y. Kim HALLA0710 (354)* (KH); Jeju-si, Chuja-myeon, Yecho-ri, Chuja-gyo-Mt. Dondaesan, 157 m, 1 Jul 2013, *S.H. Park, S.Y. Jeong, C.W. Lee, J.K. Hong P131790, P131750* (KH); Seogwipo-si, Saekdal-dong, 700 Hill, 1100 m, 28 Aug 2013, *G.Y.Chung et al. ANH-04130416* (KH); Jeju-si, Jochon-eup, Gyorae-ri, 438 m, 26 Sep 2018, *S.G. Lee 180849* (KB). **Jeollanam-do:** Korean Archipelago, 1863, *R. Oldham 846* (GH); same locality 1863, *R. Oldham 846* (P); Wando, 20 Jun 1913, *T. Nakai 523* (TI); Baekyangsan (Currently Baekamsan), 5 Jun 1928, *T. Naka, 10888* (TI); Wando, 8 Aug 1933, *S*. *Okamoto* *s.n.* (KYO); Mt. Tii., Yangseon-dong, 17 Sep 1934, *S. Okamoto 18773* (KYO); Yeongam, Mt. Wolchulsan, 25 Jun 1936, *H.D. Jang 1734* (TI); Joseon Jeollanam-do Yeongam, Mt. Wolchulsan, 25 Jun 1936, *H.D. Jan, 1734* (2 sheets) (KYO); Jangseong-gun, Buksang-myeon, Manga, 10 Jul 1960, *J.S. Le, 2014* (KH); Yeonggwang-gun, Bulgap-myeon, Bulgapsan, 1 Jul 1989, *B.Y. Sun et al*. *s.n.* (4 sheets) (KB); Wando-gun, Wando-eup, Daeya-ri, 140 m, 21 Jun 2014, *I.B. Lee, S.M. Han* *s.n.* (KB); Gurye-gun, Sandong-myeon, Manbokdae, 27 Jul 1997, *B.Y. Sun et al. s.n.* (3 sheets) (KB); Namwon-si, Cheonhwangbong, 27 Sep 1999, *B.U. Oh 25698CBU* (KH); Sinan-gun, Jindo-eup, 29 Jun 2000, *H.T. Im, H.H. Hong KNAH021197, KNAH021199* (KH); Wando-gun, Kumil-eup, Pyeonggil-do, along the road from Singu-ri to Wolsong-ri, 10–50 m alt., 9 Aug 2001, *H.T. Im 038382* (TI); Sinan-gun, Anjwa-myeon, Sangsa-chido, 10 Jun 2002, *S.H. Park, Park SH22148, Park SH22149, Park SH22154, Park SH22155* (KH); Shinan-gun, Sangseochi-do, 10 Jun 2002, *J.H. Kim 058* (KB); Jindo-gun, Gogun-myeon, Gambu Island, 20 Jun 2002, *J.O. Hyun, H.K. Park* *s.n.* (2 sheets) (KB); Sangsa-chido, 11 Jun 2002, *E.S. Jeon* *s.n.* (6 sheets) (KH); Haenam-gun, Bukpyeong-myeon, Mt. Durunsan, Jinbulam Valley, 8 Jun 2002, *S.H. Park Park SH22198* (KH); Jindo-gun, 21 Jun 2002, *J.O. Hyun, H.K. Park 2002141* (KH); Jindo-gun, Gunnae-myeon, Narisan, 213 m, 14 Jun 2003, *E.S. Jeon* *s.n.* (KH); Wando-gun, Gogeum-myeon, Saemosil, Mt. Deokamsan, 180 m, 6 Aug 2003, *J.G. An, Y.J. Yoon, 0380600806* (KB); Wando-gun, Gogeum-myeon, Saengil-myeon, Seoseng-ri, Mt. Baekunsan, 467.6 m, 10 Aug 2003, *J.G. An, Y.J. Yoon 0381000333* (KB); Jangheung-gun, Jangheung-myeon, Hasan-ri, Mt. Jeamsan, 17 Oct 2003, *J.G. An, Y.J. Yoon s.n.* (2 sheets) (KB); Jangheung-gun, Gwansan-eup, Cheonwansan, 24 Jun 2003, *J.G. An, M.N. Seol s.n.* (KB); Gwangju City, Wolgaksan, 27 Jun 2004, *Y.S. Kim D-088* (KB); Hampyeong-gun, Hampyeong-eup, Japoong-ri, Gambangsan, 257.2 m, 25 Jun 2005, *s.n.* (KB); Jangseong-gun, Eunseon-dong, Ipamsan, 15 Jul 2005, *H.C. Lee J.G. An, Y.N. Yoo, S.M. Moon 10082* (3 sheets) (KB); Gokseong-gun, Gokseong-eup, Tongmyeongsan Yongge Village Valley Forest Road, 232 m, 26 Oct 2005, *G.H. Nam NGH50423* (KH); Wando-gun, Nohwa-eup, Somasakdo, 28 Jun 2006, *H.T. Im, H.D. Son 0657611, 065760* (KB); Wando-gun, Nohwa-eup, Somasakdo, 1 Jul 2007, *H.T. Im 073226* (KB); Sinan-gun, Docho-myeon, Oryu-ri, Simok Beach, Back Mountain, 30 m, 24 Oct 2007 *Y.H. Cho, D.S. Ryu, J.G. Lee, S.J. Choi, O.S. Kim WR-071024-171* (KH); Haenam-gun, Hwaeon-myeon, Husan-ri, Deunggae Village hillside, 50 m, 10 Oct 2007, *Y.H. Cho, D.S. Ryu, J.G. Lee, S.J. Choi, O.S. Kim WR-071010-027* (KH); Goheung-gun, Gwayeok-myeon, Yeondeung-ri, Mideokdo, 25 Jun 2008, *H.T. Im E08004* (KB); Goheung-gun, Geumsan-myeon, Gheon-ri, Daechwido, 60 m, 24 Jun 2008, *G.Y. Jeong*, *M.S. Park* *s.n.* (2 sheets) (KB); Goheung-gun, Bongrae-myeon, Soryeong-do, 26 Jun 2008, *D.K. Kim, S.H. Hwang 251* (KB); same locality, 26 Jun 2008, *D.K. Kim, S.H. Hwang 113* (KB); Jindo-gun, Gunnae-myeon, 30 Jun 2008, *S.H. Park, J.C. Yang* *s.n.* (3 sheets) (KH); Sinan-gun, 24 Oct 2007, *Y.H. Cho NAM-071024-200* (KH); Sinan-gun, Aphae-do, Mt. Songgongsan, 69 m, 17 Jul 2009, *E.S. Jeon E.S.Jeon90620* (KH); Yeosu-si, Sinwol-dong, Neungcho-do, 10 m, 24 Jun 2009, *H. Kim HKim5088* (KB); Sinan-gun, Aphae-myeon, Bokyong-ri, Gemsan, 1 Jul 2009, *H.D. Son, J.G. Seo* *346021-0074* (KB); Yeosu-si, Hwajeng-myeon, Nangdo-ri, 22 Jun 2009, *D.G. Kim, S.J. Jo* *s.n.* (KB); Sinan-gun, Aphae-myeon, Songgong-ri, Songgongsan, 11 Jun 2009, *H.D. Son, J.G. Seo* *s.n.* (KB); Goheung-gun, Geumsan-myeon (Geumgumdo), Pasongjae-Yaksu, 6 Jul 2010, *Y.J. Chung Goheung (Geumgumdo)-100706-035, Chung Goheung (Geumgumdo)-100706-036* (KH); Sinan-gun, Jido-eup, Gitdaebong (Bukgye-guk ridge-top), 40 m, 18 Jun 2010, *J.C. Yang, G.S. Jang, H.S. Yoo, J.R. Jeong Y100544* (KH); Goheung-gun, 24 Jun 2010, *S.H. Kang et al.* *Goheung (Geumgumdo)-100624-068* (KH); Wando-gun, Geumdang-myeon, Geumdang-do, 127 m, 17 Jun 2010, *S.C. Ko, D.C. Son, D.S. Choi MINHM-21010-0542* (KH); Sinan-gun, Anjwa Island, 2 Jul 2010, *W.K. Paik 100702-270* (KH); Sinan-gun, Sinui-myeon, Sangtae-seori, Sogi-do, 22 m, 21 Jun 2010, *H. Kim, S.J. Kwon, C.H. Lee HKim5530* (2 sheets) (KB); Haenam-gun, Hwasan-myeon, Gwangdong-ri, Seongjwa-do, 177 m, 13 Jun 2010, *H.T. Im Im 50840* (KB); same locality, 150 m, 13 Jun 2010, *H.D. Son* (KB); Sinan-gun, Aphae-myeon, Songgong-ri, Songgongsan Wetland, 7 Jul 2010, *H.D. Son Im50357* (KB); Sinan-gun, Anjwa-myeon, Hanun-ri, Mundong-do, 22 Jul 2010, *H.T. Im, H.D. Son s.n.* (KB); Sinan-gun, Bigeum-myeon, Dogori, Biaseom, 21 Jun 2010, *D. Kim, J.H. Cho* *s.n.* (KB); Sinan-gun, Jangsan-myeon, Majin-do-ri, Gudo, 20 Jun 2010, *H.T. Im, H.D. Son s.n.* (KB); Sinan-gun, Anjwa-myeon, Jara-ri, Seungdo, *H.T. Im, H.D. Son s.n.* (KB); Sinan-gun, Jangsan-myeon, Oeum-ri, Seodonaegi, 21 Jun 2010, *H.T. Im, H.D. Son s.n.* (KB); Wando-gun, Geumdang-myeon, Junghwa-do, 29 m, 11 Jul 2011, *W.B. Seo, B.W. Han s.n.* (2 sheets) (KB); Jindo-gun, Jodo-myeon, Sinjeon-ri, Sinjeon Beach, 28 m, 28 Jul 2011, *J.W. Jang, H.J. Lee, H.S. Hwang s.n.* (2 sheets) (KH); Sinan-gun, Aphae-myeon, Gori, Haedu Island, 21 Jun 2011, *J.W. Jang, E.M. Sun s.n.* (3 sheets) (KB); Wando-gun, Geumi-eup, cheokchi-ri, coastal area, 1 Oct 2011, 36 m, *J.G. An, Y.J. Yoon 347093-50440* (KB); Wando-gun, Geumdang-myeon, Yuksan-ri, next to the reservoir, 25 m, 13 Aug 2011, *J.G. An, Y.J. Yoon 347091-50058* (2 sheets) (KB); Sinan-gun, Jungdo-myeon, Bangchuk-ri, Okdo, 20 Jun 2011, *H.T. Im, J.W. Jang, E.M. Sun s.n.* (KB); Wando-gun, Geumdang-myeon, Yuksan-ri, Junghwa-do, 11 Jul 2011, *J.O. Hyun, B.W. Won, W.B. Seo s.n.* (2 sheets) (KB); Sina-gun, Aphae-myeon, Janggam-ri, Odo, 23 Jul 2011, *H.T. Im, J.W. Jang, E.M. Sun s.n.* (3 sheets) (KB); Jindo-gun, Imhoe-myeon, Namdong-ri, Yeongwisan, 77 m, 1 Jul 2012, *B.W. Han, W.B. Seo s.n.* (2 sheets) (KB); Jindo-gun, Imhoe-myeon, Namdong-ri, Yeongwisan, 53 m, 10 Aug 2012, *G.Y. Byeon* (KB); Jindo-gun, Gunnae-myeon, Yongjang-ri, Yongjangsanseong Fortress, 35 m, 29 Jun 2012, *U.N. Kwon* *s.n.* (KB); Jindo-gun, Jindo-eup, Sinwol-ri, 25 m, 1 Jul 2012, *K.L. Shin* *s.n.* (KB); Jindo-gun, Gogeun-myeon, Byeokpari, 228 m, 29 Jun 2012, *B.W. Han, W.B. Seo s.n.* (2 sheets) (KB); Sinan-gun, Aphae-myeon, Bokyeong-ri, 32 m, 20 Aug 2012, *H.J. Kwon, B.C. Lee, W.B. Seo, A.N. Seo* *s.n.* (KB); Sinan-gun, Aphae-myeon, Daecheon-ri, 41 m, 5 Oct 2012, *H.J. Kwon* *s.n.* (KB); Jindo-gun, Imhoe-myeon, Namdong-ri, Jilmae-dong, 43 m, 28 Jun 2012, *B.W. Han, W.B. Seo s.n.* (KB); Sinan-gun, Bigeum-myeon, Gwangdae-ri, Seongchisan, 65 m, 13 Aug 2012, *H.S. Won, S.H. Baek 345044-85* (2 sheets) (KB); Sinan-gun, Imja-myeon, Iheukari, 11 Jun 2012, *E.S. Jeon* *E.S.Jeon356133-0460* (2 sheets) (KB); Wando-gun, Saengil-myeon, Bongseon-ri, Mokseom, 27 Jul 2012, *H.T. Im, H.D. Son s.n.* (KB); Sinan-gun, Palgeum-myeon, Danggori Gosan, 13 Jun 2012, *E.S. Jeon E.S.Jeon346014-0684, E.S.Jeon346014-0406* (KB); Hwasun-gun, Nammyeon, Jangjeon-ri, Sapyeong Wetland, 136 m, 25 Jul 2012, *Y.H. Cho WR-20120725-003* (KH); Jindo-gun, Jisan-myeon, Eunmuri, Mt. Jiryeoksan, 230 m, 30 Jun 2012, *s.n. 201106-464* (KB); Jindo-gun, Imhoe-myeon, Yeondong-ri, Hanboksan, 68 m, 15 Jun 2013, *G.A. Jeong, O.Y. Cho, J.Y. Lee JJP4065* (KB); Sinan-gun, Aphae-do, 34 m, 11 Sep 2014, *Y. Lim, G.P. Yoo 2014JNV030* (KH); Docho-myeon, Suhang-ra, Docho-do, 16 m, 12 Oct 2012, *Y. Lim, G.P. Yoo 2014JNV053* (KH); Hwasun-gun, Nammyeon, Jangjeon-ri, Sanji Wetland, 135 m, 26 Jul 2014, *Y.H.Cho, J.H. Kim WR-140726-052* (KH); Hwasun-gun, Hancheon-myeon, Hangye-ri, 150 m, 26 Jul 2014, *Y.M. Cho, J.H. Kim WR-140726-030* (KH); same locality, 135 m, 26 Jul 2014, *Y.M. Cho, J.H. Kim WR-140726-092* (KH); Gunsan-si, Bieungdo, 20 m, 2 Jul 2015, *M.Y. Kim, H.S. Choi 201553046* (KH); Muan-gun, Cheonggye-myeon, Cheongcheon-ri, San 113–1,149 m, 29 Jun 2016, *Y.C. Kim, H.H. Chae* *s.n.* (KB); Muan-gun, Hyeongyeong-myeon, Haeun-ri, San 105, 107 m, 30 Jun 2016, *Y.C. Kim, H.H. Chae* *s.n.* (KB); Jindo-gun, Gunae-myeon, Nokjin-ri, San 2887, 10 m, 10 Jul 2016, *Y.H. Cho, J.H. Kim, S.S. Kim, H.J. Na s.n.* (KB); Around Wando Arboretum, 10 Jul 2018, *S.G. Shin, S.J. Lee 20180710-75-02* (KH); Sinan-gun, Jaeun-myeon, Baeksan-ri, Jaeun-do, 27 m, 24 Jul 2019, *Y.M. Kim, M.S. Choi KYM190724-07* (2 sheets) (KB); Sinan-gun, Jangsan-myeon, Dasuri, 14 m, 19 Jun 2019, *B.U. Han, S,C, Yu, S. Moon s.n.* (3 sheets) (KB); Sinan-gun, Palgeum-myeon, Wonsan-ri, Palgeum-do, 11 m, 25 Jul 2019, *Y. Kim, M.S. Choi* (2 sheets) (KB); Sinan-gun, Amtaemyeon, Osan-ri, Amtaedo, 4 m, 17 Jun 2019, *B.W. Han, S.C. Yu, S. Moon* *s.n.* (3 sheets) (KB); Sinan-gun, Bigeum-myeon, Bigeum-do, 2 Jun 2021, *J.S. Lee LJS201602-1* (KB): Sinan-gun, Aphae-eup, Aphae-do, Bokryeong-ri, Holmoesan, 50 m, 14 Jul 2023, *H.T. Im, S.Y. Park* *s.n.* (KB); Sinan-gun, Sinui-myeon, Sangtae-seori, 7.2 m, 26 Aug 2024, *J.S. Lee LJS24-372* (KB). **Jeollabuk-do:** Mt. Tii., Below Mt. Sambongsan, 18 Jul 1937, *S. Okamota s.n.* (KYO); Gunsan-si, Okdo-myeon, Seonyudo, 24 Sep 1982, *J.S. Park, D.U. Kim s.n.* (KB); Jeonju-si, Wansan-gu, Jungni-dong, Mt. Moaksan, 27 Jun 1987, *C.H.Kim* *s.n.* (KB); Buan-gun, Jinseo-myeon, Seokpo-ri, Naesosa Temple, Naebyeonsan, 1 Jul 1990, *S.Y. Lee* *s.n.* (2 sheets) (KB); Buan-gun, Jinseo-myeon, Baekcheonnae, 15 Aug 1991, *G.J. Ko, S.Y. Lee* *s.n.* (KB); same locality, 15 Aug 1991, *H.Y. Lee, H. J. Kim* *s.n.* (KB); same locality, 16 Aug 1991, *E.H. Seo, H.J. Kim* *s.n.* (KB); Wanju-gun, Dongsang-myeon, Mt. Unjangsan, 75 m, 15 Jul 1993, *J.S. Lim* *s.n.* (KB); Wanju-gun, Dongsang-myeon, Unjangsan, 20 Jul 2001, *B.Y. Sun* *s.n.* (KB); Gunsan City, Okdo-myeon, Shinsido, 29 Jul 2003, *C.H. Kim et al. s.n.* (KB); Jangsu-gun, IOC. M.T. Yonggu, 2 Jul 2004, *W.K. Paik Jangsu-gun (Yonggusan)-040702-156* (KH); same locality, 632 m, 18 Sep 2004, *W.K. Paik* *Jangsu-gun (Yonggusan)-040918-296, Jangsu-gun (Yonggusan)-040918-295* (2 sheets) (KH); Buan-gun, Sangseo-myeon, Ugeumsanseong Fortress, 74.6 m, 30 Jun 2004, *G.Y. Jeong Buan-gun (Ugeumsanseong)-040630-038* (KH); Gunsan-si, Okdo-myeon, Dan-eum-do, 22 m, 25 Jul 2007, *G.Y. Jeong, M.S. Park s.n.* (KH); same locality, 25 Jun 2007, *G.Y. Jeong, M.S. Park s.n.* (KB); Gunsan-si, Okdo-myeon, Seonyudo-ri, Seonyudo, 151 m, 19 Jul 2012, *S.H. Park, C.W. Lee H.J.Lee, J. Jang ParkSH122446* (KH); same locality, Seonyubong, 152 m, 19 Jul 2012, *S.H. Park, C.W. Lee H.J.Lee, J. Jang ParkSH122447* (KH); same locality, Munyeodoli, Munyeobong the mountain behind, 78 m, 18 Jul 2012, *S.H. Park, C.W. Lee H.J. Lee, J. Jang ParkSH122376* (2 sheets) (KH); Gunsan-si, Okdo-myeon, Shinsido, North slope of Daegaksan, 117 m, 11 Oct 2012, *S.H. Park Park SH 124761* (KH); Sunchang-gun, Sangchi-myeon, Unam-ri, San 28–1, 284 m, 26 Jul 2016, *Y.C. Kim, H.H. Chae* *s.n.* (KB); Goheung-gun, Bongrae-myeon, Oenaro, Mt. Bongraesan, 20 m, 23 Jul 2016, *J.G. An, S.H. Hwang* *s.n.* (KB); Gunsan-si, Okdo-myeon, Shinsido-ri, Wolyeongbong, 136 m, 29 Jun 2016, *G.U. Lee, J.H. Cho* *s.n.* (4 sheets) (KB); Gunsan-si, Okdo-myeon, Yamidori, 56 m, 30 Jun 2016, *J.H. Kim, J.H. An s.n.* (2 sheets) (KB). **Chungcheongbuk-do:** Mungyeong-si, Sanbuk-myeon, Mt. Gongdeoksan, 451 m, 7 Jul 2007, *G.Y. Chung ANH-0207045* (KH); Eumseong-gun, Gwanghyewon-myeon, Mt. Deokseongsan, 10 Aug 2004, *J.H. Kim, J.B. Lee s.n.* (KB). **Chungcheongnam-do:** Seosan, Wonbuk-Myeon, 1.5 km NW of Taesung Yum Jun (salt pans), 25 Aug 1984, *B.R. Yinger, Y.J. Chang, G.M. Sylvester, D.A. Apps, W.B. Peters, W.M. Paul 2612 s.n.* (MOAR); Dangjin-gun, Yumo-do, 27 Jul 2001, *E.S. Jeon* *s.n.* (KH); Seosan-si, Palbong-myeon, Mt. Palbongsan, 29 Sep 2006, *H.J. Choi CHU60701* (2 sheets) (KH); Seocheon-gun, Seomyeon, Oknyeo Peak, 2 Jul 2005, *S.H. Yeo 20580* (KB); same locality, 21 Aug 2005, *S.H. Yeo 20471* (KB); Seosan-si, Heukeodo, 13 m, 24 Aug 2006, *H. Kim HKim 2666* (3 sheets) (KB); Taean-gun, Geunheung-myeon, Sinjin-do-ri, 74 m, 6 Jul 2011, *S.C. Ko, Y.H. Hong, D.C. Son s.n.* (2 sheets) (KB); Taean-gun, Sowon-myeon, Mohang-ri, 18 m, 24 Jun 2014, *B.W. Han, W.B. Seo s.n.* (3 sheets) (KB); Taean-gun, Sowon-myeon, Mohang-ri, 13.6 m, 6 Aug 2024, *J.S. Lee LJS24-373* (KB); Taean-gun, Sowon-myeon, Mohang-ri, Haenggeum Beach Road, 43 m, 4 Jul 2024, *J.S. Lee LJS24-374, LJS24-375* (KB); Taean-gun, Wonbuk-myeon, Hwangchon-ri, 24 m, 3 Jul 2024, *J.S. Lee LJS24-376, LJS24-377* (KB). **Gyeonggi-do:** Ansan-si, Danwon-gu, Daebubuk-dong, Gubong-do, 25 m, 19 Aug 2011, *S.H. Park, S.Y. Jeong, J. Jang, H.S. Hwang ParkSH113238* (KH); Ansan-si, Danwon-gu, Daebudong, Gubongdo, 49 m, 14 Jul 2016, *G.H. Nam, H.G. Oh, J.G. Hong s.n.* (2 sheets) (KB). **Incheon:** Mt. gaeyang, 16 Jun 1986, *C.B. Shin 001106* (KH); Ongjin-gun, Daecheong-myeon, Daecheong-do, 14 Aug 2008, *Y.M. Lee, J.C. Yang Y80706* (KH); Ongjin-gun, Baekryeong-myeon, Jinchon-ri, Baekryeong-do, 91 m, 10 Jul 2012, *J.H. Kim, M.S. Lee, J.S. Kim BN700* (KB); Ongjin-gun, Baekryeong-myeon, Jincheol-ri, Baekryeong-do, 41 m, 11 Oct 2023, *J.H. Kim, S.U. Kwon KIMJH23247* (KB).

**China.** **Zhejiang:** Shaoxing City, Xinchang County, Tianmu Mountain, Mountains, ditch side, wet soil, 520 m, 18 Jun 1958, *Anonymous 305* (HZ); same locality, 18 Jun 1958, *Anonymous 305* (NAS); same locality, 800 m, 26 Jun, *Anonymous 2046* (HZ); Taizhou City, Tiantai County, Tiantai Mountain, *s.d.*, *s.n. 0305* (PE).

**Japan.** **Honshu:** Tokyo, Ome, Mitaki-san, Musashi, Jul 1891, *K. Watanabe* *s.n.* (GH); Montagne Yamagata, *s.d.* *U.J. Faurie 4307* (AMES); *s.l.* Aug 1899, *U.J. Faurie 4608* (AMES); Miyagi, Sendai, 25 Sep 1916, *A. Yasuda* *s.n.* (AMES); Mino Region, Korigami Town, Futsukamachi, 24 Jul 1921, *K. Shiota 2203* (AMES); Mie-ken, Watarai-gun, Natsukusa, 27 Jun 1929, *R.K. Beattie,* *Y. Kurihara 10887* (GH); Mino Region, Kano Town, Ota, 4 Jul 1931, *K. Shiota 5123* (AMES); Mino Region, Korigami Village, Takawashi, 7 Jul 1932, *K. Shiota 6264* (AMES); Mino Region, Taki Town, Tsumagi, 25 Jul 1934, *K. Shiota 8125* (AMES); Mino Region, Toki Town, Shitaishi, 23 Aug 1935, *Y. Kato 9379* (AMES); Mino Region, Toki Village, Suazu, 3 Aug 1935, *s.n. 9228* (AMES); Rokkosan vic. Kobe Honsyu Nippon, 12 Jul 1936, *K. Uno 14516* (AMES); Kyoto, Kyoto-fu Nippon, 20 May 1937, *K. Uno 18752* (AMES); Chiba, Sanbu-gun, Sanbu-cho, Hanita, 1939, *T. Makino 192719* (KYO); Kanagawa, Near the Hakone Shrine, Motohakone, 700 m, 7 Jul 1948, *H. Kanai 6091* (A); Hiroshima, Fort of Dogoyama, 25 Jul 1949, *K. Uno 212609* (AMES); Gumma, Kwanto District, West Kuro-dani Valley about 9 km NW of Doai, 25 Aug 1951, *T. M. Koyama 1021* (A); Tsukute in Mikawa, 16 Oct 1954, *J. Ohwi, T.M. Koyama 1126* (GH): Takarazuka in Settsu, 29 Jun 1955, *M. Togasi 1182* (GH); Kanagawa, Sengoku-bara Hakone Ashigarashimo-gun, 15 Jul 1960, *M. Furuse* *s.n.* (A); Nagano, Shinano, foot of Mt. Surikogi Ohdaira Ihida-shi, 27 Jul 1961, *M. Furuse* *s.n.* (A); Shiga, route from Mt. Hakodate-yama to Hira-ike, Imazu-cho, Takashima-gun, 500–650 m, 23 Jul 1974, *N. Kurosaki 6326* (A); Shizuoka, Ohnoga-hara, Gotenba City. SE foot of Mt. Fuji, ca. 700 m, 15 Jul 1977, *S. Ikeya F.K 12310* (A); Nara, Nara-shi, Ninnikusen, 13 Jul 1979, *M. Ito, E. Kinoshita 69* (A); Iwate, Hanamaki-shi, Nishi-Miyanome, Kimigasawa, Hanatsutsumi, 96 m, 16 Jul 1984, *A. Takehara 200* (A); Miyagi, Sendai-shi, Aoba-ku, Aramaki, Nitayachi, 110–120 m, 17 Jul 1992, *K. Yonekura 650* (A). Kyushu: Nagasaki, 1862, *R. Oldham 247* (GH); Kumamoto-hyen, IOC. Mt. Aso, 1,050 m, 7 Jul 2004, *W.K. Paik Kyushu2-040707-044* (2 sheets) (KH). **Hokkaido:** Japonia Hakodate, 1861, *C.J. Maximowicz s.n.* (GH); Southern Hokkaido, 1884, *W.P. Brooks 591* (AMES); Oshima; at the vicinity of Yunokawa-cho, Hakodate-city, 10 m, 30 Jul 1969, *H. Takahashi 505* (TI).

**2. *Epipactis xanthophaea*** Schltr.

**Korea.** **Jagang-do:** Huchang-gun, 15 Aug 1897, *V.L. Komarov s.n.* (LE). **Hamgyeongbuk-do:** Gyeongseong, 13 Jul 1930, *J. Ohwi s.n.* (KYO). **Gangwon-do (North):** Pyeonggang-gun, Sepo Wetland, 13 Jul 1915, *s.n.* (2 sheets) (TI); Jangjeon, Geumgangsan area, 29 Aug 1916, *T. Nakai 6031* (TI). **Gangwon-do:** Yeongwol-gun, Suju-myeon, 5 Jul 2014, *Y.S. Oh, S. Mun, G.Y. Lee YR07A1034* (KB); Yeongwol-gun, Mulmuri Valley, 19 Aug 2020, *J.S. Lee LJS200819-1* (KB); Inje-gun, 716 m, 2 Jul 2024, *J.S. Lee LJS24-389* (2 sheets) (KB); Yeongwol-gun, Yeongwol-eup, San 131-1, 248 m, 4 Jul 2024, *J.S. Lee LJS24-390* (2 sheets) (KB); same locality, 290 m, 4 Jul 2024, *J.S. Lee LJS24-399* (2 sheets) (KB); Yanggu-gun, Dongmyeon, 638 m, 6 Jul 2024, *J.S. Lee LJS24-391* (2 sheets) (KB); Yeongwol-gun, Yeongwol-eup, 248 m, 6 Aug 2024, *J.S. Lee LJS24-392* (KB). **Gyeongsangnam-do:** Sancheong-gun, Samjang-myeon, 30 Jun 2004, *J.M. Chung, J.Y. Kim 040603211* (KH); Sancheong-gun, Samjang-myeon, 987.1 m, 19 Sep 2024, *J.S. Lee LJS24-394* (3 sheets) (KB). **Jeollabuk-do:** Muju-gun, Mt. Deogyusan, 20 Jun 2012, *H.S. Choi, M.Y. Kim JB-V2012156* (KH). **Jeollanam-do:** Okgwa-gokseong, 28 Jun 1913, *T.* *Nakai s.n.* (TI). **Incheon:** Jung-gu, San 213, 28.1 m, 4 Sep 2024, *J.S. Lee LJS24-393* (KB).

**China.** **Heilongjiang:** Mandschuria, 19 Jul 1896, *V.L. Komarov s.n.* (LE); Growing on a hillside meadow, 3 Sep 1959, *Y. Zhang et al. 2100* (PE); Jixi, Hulin, 15 Jul, *s.n. 085* (HNWP). **Jilin:** Mandschuria, 18 Jul 1896, *V.L. Komarov 451* (LE); Mandschuria, 22 Aug 1896, *V.L. Komarov s.n.* (LE); Sze-chi-teng, Ching-po-lake, marshy place, 300 m, 2 Aug 1931, *F.H. Chen 253* (3 sheets) (PE); same locality, 2 Aug 1931, *F.H. Chen 253* (3 sheets) (AMES); China, Dunhua, Aekmok, 21 Aug 2009, *D.K. Lee 2009-0707* (2 sheets) (KH); same locality, 6 Jul 2010, *D.K. Lee 2010-056-4* (3 sheets) (KH); Dunhua, Aekmok Wetland, 10 Jul 2012, *J.O. Hyun NAPI-2012-0600* (NIBR). **Liaoning:** Zhangwu County, Yang Family's Small Pond in Zhanggutai, 1 Jul 1953, Z. *Wang, Z,* *P. Fu et al., 2513* (3 sheets) (HNWP). **Shandong:** Kunyu Mountain, 10 Jul 1956, *s.n. 2991* (PE); Dezhou City, Dushan Mountain, In the moist grassland on the hillside, it often coexists with irises, 17 Jun 1959, *T. Zhou et al. 2289* (PE); Yantai, Haiyang, Zhao Hu Mountain, In the grass on the hillside, 20 Jun 1959, *T. Zhou et al. 2309* (PE); Weihai City, Wendeng County, Lao Huai Bridge, 2 Jul 1960, *s.n. 31* (4 sheets) (PE).

**Russia.** Primorsky Krai, 19 Jul 1950, *G.K. Smirnova 590* (2 sheets) (MHA); Kirovskiy village, in the swamp near the “Shmakovka” mineral spring, *P. Torobots, N. Thrzenzob s.n.* (A); Primorsky Krai, 13 Aug 1978, *G.A. Belova* *s.n.* (MHA); Siberia, Russian Far East (S6) , 13 Aug 1978, *T.M. Lapteva* *s.n.* (MHA); Primorsky Krai, 22 Jul 1980, *O.* *Koval s.n.* (MHA); Khasansky District, Primorsky Krai: 'Kedrovaya Pad.' Primorsky Meadow, 30 Jul 1985, *s.n.* (MW); Primorsky Krai, Khasansky District, 5km south of the settlement Primorsky, 12 Aug 1986, *V.* *Korotkov s.n.* (MW); Primorsky Krai, Khasansky District, Primorskaya Station area, 26 Jul 1989, *I.* *Tatarenko s.n.* (MW); Primorsky Krai, Khasansky District, Primorskaya Station area, 27 Jul 1990, *I.* *Tatarenko* *s.n.* (MW); Primorsky Krai, Khasansky District, Ryazanovka Station, near Rakushechnaya Bay, 7 Jul 1995, *I.* *Tatarenko s.n.* (MW).

**3*. Epipactis papillosa*** Franch. & Sav.

**Korea.** **Gangwon-do:** Mt. Geumgangsan, 14 Aug 1902, *T. Uchiyama* *s.n.* (TI); Mt. Odae-San, 25 Jul 1946, *C.I. Cho 3019* (MICH); Yeongwol-gun, Seomyeon, Neureupjae, 3 Jul 2003, *J.O. Hyun, H.K. Park 2003096* (3 sheets) (KH); Yeongwol, 4 Jul 2004, *Y.S. Kim D-083* (KB); Yeongwol-gun, Yeongwol-eup, Open space, 16 Sep 2004, *J.O. Hyun, H.K. Park 2004242* (2 sheets) (KH); Samcheok City, Mt. Deokhangsan, 780 m, 25 Jun 2005, *J.O. Hyun, S.C. Lee, H.J. Kwen, Y.S. Oh KTAPS20050462* (KH); Gangneung-si, Okgye-myeon, Mt. Seokbyeongsan, 760 m, 21 Jun 2006, *J.O. Hyun, H.J. Kwen, Y.S. Oh, E.K. Choi s.n.* (KH); Samcheok City, Miro-myeon, Dutasan, 389 m, 1 Jul 2007, *J.O. Hyun, H.J. Im, M.G. Im, Y.U. Gwak s.n.* (4 sheets) (KH); Hwacheon-gun, Gandong-myeon, Mt. Byeongpungsan, 16 Jul 2008, *W.K. paik Hwacheon-gun (Mt. Byeongpungsan)-080716-295* (KH); Jeongseon-gun, Mt. Nomoksan, 23, Jul 2008, *B.U. Oh Nomoksan-080723-004* (4 sheets) (KH); Taebaek-si, Changjuk-dong, Geomryongso, 931 m, 9 Jul 2008, *E,S, Jeon ESJeon80515* (KH); Samcheok-si, Singi-myeon, Machari, Yeonhwa-gyo Village, 257 m, 10 Jun 2009, *J.C. Yang, J.W. Jang, Y.J. Yoon K10012* (KH); Samcheok-si, Shingamyeon, Daeyari, Mt. Deokhangsan (Golmal-Deokhangsan), 12 Jun 2009, *J.C. Yang, J.W. Jang, Y.J. Yoon K10308* (KH); Samcheok-si, Hajangmyeon, Yongyeon-ri, Sambongsan, the top, 1,215 m, 23 Jul 2009, *C,H. Shin, S.S. Jeong, S.C. Baek,* *J.E.* Yoon K11337 (KH); Yeongwol-gun, Seomyeon, Ongjeong-ri, 328 m, 4 Jul 2009, *J.W. Yun* (KH); Samcheok-si, Gagok-myeon, Mt. Myeonsan, 280 m, 27 Jun 2009, *J.O. Hyun, J.H. Lee, H.J. Yim NI804010* (KH); same locality, 251 m, 20 Sep 2009, *J.O. Hyun, H.J. Yim, M.S. Choi 1303050* (KH); Taebaek-si, Cheoram-dong, Mt. Myeonsan, 737 m, 27 Jun 2009, *J.O. Hyun, S.I. Kang, Y.S. Jeong 803009* (KH); Yeongwol-gun, Hadong-myeon, Jinbeol-ri, 15 Sep 2010, *G.H. Nam, J.H. Park* *s.n.* (KB); Yeongwol-gun, Suju-myeon, Mureung-ri, 300 m, 3 Jul 2010, J.O. *Hyun, Y.S. Oh, G.S. Kim, J.M. Jeong, A.R. Park* *s.n.* (KB); Samcheok-si, Gagok-myeon, Punggok-ri, Seokgaejae, 4 Aug 2011, *J.S. Kim, B.C. Kang, D.J. Kim, C.W. Park, H.J. park, S.H. Jang 251SJ-12* (KB); Samcheok-si Geundeok-myeon Gyoga-ri Geunsan, 11 m, 25 Jul 2011, *Y.I. Go Go379092-114* (KB); Samcheok-si Gagok-myeon Deokpung-ri Mt. Munbangsan 912 m, 18 Jul 2011, *G.Y. Chung VP-KB-1107-0280* (KB); Jeongseon-gun, Imgye-myeon, Eumji-mal 794 hill top, Mt. Munrae, 793 m, 22 Jul 2011, *G.Y. Chung 378121-0110* (KB); Donghae-si swaeum-dong, 52 m, 21 Jul 2011, *G.H. Nam, J.Y. Lee, J.S. Kim NISHY2-1078* (2 sheets) (KB); same locality, 33 m, 8 Jun 2011, *G.H. Nam, Y.I. Yoon, Y.R. Kim NISHY2-596* (2 sheets) (KB); same locality, 52 m, 21 Jun 2011, *G.H. Nam, J.H. Kim, J.Y. Lee SHY2-624* (KB); Jeongseon-gun, Imgye-myeon, Mt. Jahusan, 874 m, 17 Jul 2011, *H.G. Lee, G.S. Kim, Y. Song 0901008* (KH); Jeongseon-gun, Hwaam-myeon, 646 m, 2 Jul 2011, *H.G. Lee, Y.S. Oh, H.J. Kim 0802007* (KH); Samcheok-si, Geundeok-myeon, Gyogari, 11 m, 25 Jun 2011, *Y.J. GO s.n.* (2 sheets) (KH); Pyeongchang-gun, Pyeongchang-eup, Hari, 386 m, 21 Sep 2011, *J.D. Lee et al.* *s.n.* (KB); Donghae-si, Cheongok-dong, 56 m, 10 Aug 2011, *J.D. Lee PWK-088* (KH); Yeongwol-gun, Seomyeon, Ongieong-ri, 302 m, 2 Aug 2012, *G.Y. Beon s.n.* (KB); Jeongseon-gun, Buk-myeon, Goyang-ri, Mt. Balonsan, 868 m, 17 Jul 2012, *J.H. Kim NISHY3-1064* (2 sheets) (KB); Jeongseon-gun, Bukpyeong-myeon, Mungok-ri Sangjeong Rock, 734 m, 14 Jun 2012, *G.H. Nam Y.R. Kim SHY3-898* (KB); Jeongseon-gun, Buk-myeon, Goyang-ri, Goyangsan, 1,160 m, 17 Jul 2012, *G.H. Nam, J.H. Park, S.J. Lee SHY3-1651* (KB); Jeongseon-gun, Imgye-myeon, Goyang-ri, Mt. Goyangsan, 625 m, 18 Jun 2012, *J.H. Kim, J.S. Kim, S.J. Lee SHY3-1309* (KB); Jeongseon-gun, Buk-myeon, Goyang-ri, Mt. Goyangsan, Goyanggol, 763 m, 10 Oct 2012, *G.H. Nam, Y.R. Kim SHY3-2079* (KB); Jeongseon-gun, Buk-myeon, Goyang-ri, Mt. Balonsan, 868 m, 17 Jul 2012, *G.H. Nam, J.S. Kim, J.S. Lee SHY3-1060* (3 sheets) (KB); Samcheok-si, Hajang-myeo, Jangjeon-ri, Mt. Jangbyeong, 1,055 m, 16 Jul 2013, *J.H. Kim, J.H.Park JBS130466* (3 sheets) (KB); Jeongseon-gun, Imgye-myeon, 720 m, 17 Jul 2013, *M.S. Choi, H.J. Yim 0903084* (KH); Inje-gun, Bukmyeon, Seoraksan, Jangsu-dae, 853 m, 31 Jul 2013, *D.G. Kim, S.J. Kwon 2013V079* (KH); Inje-gun, Bukmyeon, Hwanggye-ri, 899 m, 7 Sep 2013, *Y.C. Kim 2013-183* (KB); Jeongseon-gun, Jeongseon-eup, Gohan-eup, Gohan-ri, 16 Sep 2013, *J.D. Lee, G.D. Jeong, G.Y. Byeon* *s.n.* (KB); Jeongseon-gun, Hwaam-myeon, Hwaam-ri, Mt. Gatheesan, 908 m, 17 Jun 2014, *G.H. Nam, NGH14-183* (KB); Yeongwol-gun, Suju-myeon, Mureung-ri, Jucheon River, 274 m, 5 Jul 2014, *Y.S. Oh, S. Mun, G.Y. Lee YR07A1036* (KB); Jeongseon-gun, Sindong-eup, Bangje-ri, Mt. Duwibong Trail, 942 m, 11 Jul 2015, *Y.S. Oh, S. Mun, H.J. Kim JS08A-2028* (KB); Jeongseon-gun, Mt. Baegisan, 660 m, 7 Aug 2015, *Y.C. Kim 2015-218* (KB); Jeongseon-gun, Sindong-eup, Gasari, Mt. Jukyeon, 865 m, 15 Aug 2015, *Y.S. Oh, S.I. Kang, H.J. Kim JS10A1016* (KB); Jeongseon-gun, Nammyeon, Gwangdeok-ri, 468 m, 22 Aug 2015, *G.S. Kim, M.G. Lee JS10C1061* (KB); Jeongseon-gun, Nammyeon, Deogam-ri, San 50-1, 974 m, 13 Aug 2016, *Y.C. Kim, H.H. Chae KNC16-106* (KB); Jeongseon-gun, Nammyeon, Nakdong-ri, Mt. Baekisan, 565 m, 19 Jul 2016, *J.H. Kim, J.H. An, H.J. Park Baeki160906* (2 sheets) (KB); Jeongseon-gun, Jeongseon-eup, Gasuri, San 63-1, 7 Oct 2018, *Y.C. Kim 398* (KB); Jeongseon-gun, Hwaam-myeon, Hwaam-ri, 11 Jul 2021, *J.S. Lee LJS210711-1, LJS210711-2* (KB); same locality, 13 Jul 2021, *J.S. Lee LJS210713-1, LJS210713-2* (KB); Jeongseon-gun, Gohan-myeon, Gohan-ri, Hambaksan Forest Road, 25 Jul 2021, *J.S. Lee LJS210725-1* (KB); Jeongseon-gun, Gohan-eup, Gohan-ri, 1,320 m, 22 Jul 2024, *J.S. Lee LJS24-378* (KB); same locality, San 214, 1,425 m, 22 Jul 2024, *J.S. Lee LJS24-379* (KB); same locality, San 214-6, 994 m, 22 Jul 2024, *J.S. Lee LJS24-380* (KB); Yeongwol-gun, Sangdong-eup, Gurae-ri, San 1-35, 1,197 m, 22 Jul 2024, *J.S. Lee LJS24-396* (KB); same locality, 1,274 m, 22 Jul 2024, *J.S. Lee LJS24-397* (KB); Jeongseon-gun, Gohan-eup, Gohan-ri, San 2-140, 1,161 m, 22 Jul 2024, *J.S. Lee LJS24-381* (2 sheets) (KB); same locality, 1,177 m, 22 Jul 2024, *J.S. Lee LJS24-382* (2 sheets) (KB); Inje-gun, Bukmyeon, Hangye-ri, San 1-67, 899.3 m, 24 Jul 2024, *J.S. Lee LJS24-383* (3 sheets) (KB); Jeongseon-gun, Gohan-eup, Gohan-ri, 1,320 m, 25 Jul 2024, *J.S. Lee LJS24-384* (KB); same locality, 1,279 m, 16 Aug 2024, *J.S. Lee LJS24-385* (KB); same locality, San 214-6, 994 m, 3 Sep 2024, *J.S. Lee LJS24-386* (KB); same locality, San 214, 1320 m, 3 Sep 2024, *J.S. Lee LJS24-387* (KB); Jeongseon-gun, Gohan-eup, Gohan-ri, San 2-141, 1,261 m, 3 Sep 2024, *J.S. Lee LJS24-398* (KB). **Gyeongsangbuk-do:** Uljin-gun, Wonnam-myeon, Doeksin-ri, 423 m, 17 Jul 2007, *G.Y. Chung ANH-en-0707717-005* (KB); Cheongsong-gun, Budongmyeon, Mt. Juwangsan, 790 m, 12 Jul 2008, *J.O. Hyun et al. s.n.* (KH); Ulleung-gun, Seomyeon, Taehari, 76 m, 8 Jul 2009, *J.M. Jeong, Y.J. Seol, J.K. Shin JMC10869* (KH); Bonghwa-gun, Myobong, 20 Jul 2012, G.Y. *Chung et al. Bonghwa-gun (Myobong)-120720-101* (KH); Bonghwa-gun, Seokpo-myeon, 730 m, 20 Jul 2013, *G.Y. Chung et al.* *s.n.* (KB); Uljin-gun, Wonnam-myeon, Maehwa-ri, Mt. Namsusan, 5 Aug 2014, *K.S. Jeong, W. Lee, D.H. Jang JKS14411* (KB); Yeongyang-gun, Ibammyeon, Veondang-ri, Mt. Muisan, 274 m, 14 Jul 2020, *J.H. Kim, S.R. Lee YY2020893* (2 sheets) (KB); Cheongsong-gun, Budong-myeon, Buil-ri, San 157, 345 m, 26 Jul 2020, *W.J. Lee, Y.S. Lee, Y.J. Jeong CS20103* (KB); Yeongyang-gun, Ibammyeon, Yeonday-ri, Mt. Muisan, 256 m, 16 Jun 2020, *J.H. Kim, S.R. Lee, W. Lee YY2020676* (KB); Cheongsong-gun, Cheongsong-eup, Songsaeno-ri, 304 m, 5 Jun 2019, *M.Y. Lee, U.S. Choi, Y.S. Jeong CS190528* (KB); same locality, 310 m, 30 Jun 2019, *M.Y. Lee, U.S. Choi, Y.S. Jeong CS190670* (KB); Yeongyang-gun, Ibammyeon, Sinsa-ri, Buyongbong, 101 m, 10 Jul 2019, *J.H. Kim, H.J. Park Buyong 190543* (KB); Yeongyang-gun, Ibammyeon, Yeondang-ri, Mt. Buyongbong, 327 m, 11 Jul 2019, *J.H. Kim, H.J. Park Buyong 190567* (KB); Sangju-si, Hwabuk-myeon, Jangam-ri, Mt. Sokrisan Munjangdae, 29 Aug 2019, *J.S. LEE LJS190829-1* (KB). **Chungcheongbuk-do:** Gomyeong-ri, Mt. Dunusan, 272 m, 22 Jun 2012, *H.Y. Lee, S.H. Park, M.H. Song J07A3015* (KB); Jecheon-si, Susan-myeon, Dabul-ri, Samusan, Daburam, 467 m, 30 Jun 2015, *E.H. Jung, J.H. Kim, S.Y. Kim SAMU150753* (2 sheets) (KB); Jecheon-si, Susan-myeon, Goegok-ri, Mt. Samusan, 299 m, 22 Sep 2015, *E.H. Jung, J.H. Kim, M.S. Lee Samu151377* (KB); Jecheon-si, Susan-myeon, Dabul-ri, Mt. Samusan, Daburam, 435 m, 20 Oct 2015, *J.H. Kim, J.K. Hong Samu151550* (KB); Jecheon-si, Susan-myeon, Goegok-ri, Mt. Samusan, 225 m, 30 Jun 2015, *E.H. Jung et al. SAMU150715* (KB). **Chungcheongnam-do:** Geumsan-gun, Boksu-myeon, Baegam-ri, 93-1, 273m, 18 Jul 2020, *U.H. Eom CH201420* (KB). **Gyeonggi-do:** Gwangju-si, Namhansanseong-myeon, Sanseong-ri, Mt. Namhansan, 415 m, 20 Jun 2019, *J.H. Kim KimJH19052* (2 sheets) (KB); same locality, Mt. Cheongnyang, 5 Jun 2019, *S.W. Kwon KSW190037* (KB). **Incheon:** Ongjin-gun, Daecheong-myeon, Daecheong-do, Seochakjang-Dokbawi, 30 Jul 2010, *W.B. Cho, F.S. Choi 100730-1055* (KH); Ongjin-gun, Baekryeong-myeon, Jinchon-ri, Baekryeong, 81 m, 11 Jul 2012, *J.H. Kim, J.S. Kim BN 906* (2 sheets) (KB); Ongjin-gun, Baekryeong-myeon, Jinchon-ri, Baekryeongdo, Simcheong-gag, 66 m, 21 Jul 2014, *J.H. Kim, J.K. Hong KimJH14011* (KB); Ongjin-gun, Baekryeong-myeon, Nampo-ri, Baekryeongdo, 33 m, 17 Aug 2023, *S.C. Kim, S.w. Kwon KIMJH23125* (3 sheets) (KB); Jung-gu, Muui-dong, San 10-12, 27.3 m, 3 Sep 2024, *J.S. Lee LJS24-388* (KB).

**China.** **Jilin:** Baishan City, Jiangyuan County, Wanguo Town, 7 Sep 2018, *Ca. Changqing CaoChQ441* (KUN). **Liaoning:** Anshan City, Qianshan District, Wulong Mountain, 27 Aug 1959, *W. Wei et al. 1177* (IFP); The mountain is hidden beneath the dense forest trees, 10 Sep 1959, *W. Wei et al. s.n.* (IFP); Liaoning Province, 23 Aug 1964, *C. Shunchang 196* (IFP).

**Russia.** Sakhalin Oblast, 3 Aug 1908, *N.A. Palcevskij* *s.n.* (2 sheets) (AMES); Primorsky Krai, Lazovskii raion, Lazovskii, 8 Jul 1944. *P. Zhudova, Pokrovskaya s.n.* (2 sheets) (MW); same locality, 14 Aug 1945, *P. Zhudova, Pokrovskaya s.n.* (2 sheets) (MW); Primorsky Krai, Lazovskii raion, 10 Jul 1945, *P. Zhudova* *s.n.* (MW); Primorsky Krai, 12 Sep 1950, *Voroshilov* *s.n.* (MHA); Khabarovsk Krai, 24 Jul 1974, *A.A. Nechaev* *s.n.* (MHA); Khabarovsk Krai, 21 Aug 1958, *Voroshilov* *s.n.* (MHA); Sakhalin Oblast, 26 Sep 1960, *E. Egorova* *s.n.* (MHA); Kamchatka Krai, 25 Jul 1967, *A. Hohryakov* *s.n.* (2 sheets) (MHA); Khabarovsk Krai, 13 Aug 1969, *Maximova* *s.n.* (MHA); Primorsky Krai, Kavalerovskii raion, 28 Jul 1975, *Kochetkova, Pospelova* *s.n.* (MW); Sakhalin Oblast, 25 Aug 1976, *L.M. Alexeeva* *s.n.* (2 sheets) (MHA); Khabarovsk Krai, 5 Aug 1977, *Glamazdina* *s.n.* (MHA); Sakhalin Oblast, 26 Jul 1978, *A.M. Chernyaeva* *s.n.* (2 sheets) (MHA); Sakhalin Oblast, 28 Jul 1978, *E. Egorova* *s.n.* (2 sheets) (MHA); Khabarovsk Krai, Ayano-Maiskii raion, Nelkan village area, left bank of the Maya River, Chouysky lakes, 27 Jul 1978, *S. Harkevich, T. Buch* *s.n.* (MW); Khabarovsk, Ayano-Mayskiy district, vicinity of Nelkan village, left bank of Maya river, Chouysky lakes. 27 Jul 1978, *S. Sigismund, Kharkevic, T. Buch* *s.n.* (MO); Khabarovsk Krai, Ayano-Maiskii raion, 27 Jul 1978, *S. Kharkevich* *s.n.* (MHA); Khabarovskiy territory, Ayano-Mayskiy district, vicinity of the Nelkan village, left bank of Maya river, Chouysky lakes, Larix cajanderi forest, 27 Jul 1978, *Kharkevich S.,T. Buch* *s.n.* (BR); Kamchatka Krai, 8 Aug 1978, *T. Sofeykova* *s.n.* (2 sheets) (MHA); Khabarovsk Krai, Komsomol'skii raion, 18 Aug 1978, *M.S. Ignatov s.n.* (MW); Sakhalin Oblast, Holmskii raion, 10 Aug 1978, *M.S. Ignatov* *s.n.* (MW); Sakhalin Oblast, 5 Sep 1978, *E. Egorova* *s.n.* (MHA); Khabarovskiy Ayano-Mayskiy Nelkan, 27 Jul 1978, *S.Kharkevich, T.Buch 903* (KB); Primorsky Krai, 15 Aug 1983, *N.A. Shaul'skaya* *s.n.* (MHA); Primorsky Krai, 23 Jul 1985, *I.A. Shantser* *s.n.* (MHA); Primorsky Krai, Terneiskii raion, 26 Jul 1985, *I. Dorofeeva* *s.n.* (MW); Primorsky Krai, 15 Aug 1985, *I.A. Shantser* *s.n.* (MHA); Sakhalin Oblast, Korsakovskii raion, 1 Aug 1985, *V. Pavlov* *s.n.* (MW); Sakhalin Oblast, Korsakovskii raion, 16 Aug 1986, *N. Shvedchikova s.n.* (MW); same locality, 20 Aug 1986, *N. Shvedchikova* *s.n.* (MW); Kamchatka Krai, 3 Aug 1986, *T. Smirnova* *s.n.* (MHA); Sakhalin Oblast, Korsakovskii raion, 16 Aug 1986, *N. Shvedchikova s.n.* (MW); Sakhalin Oblast, Uzhno-Kuril'skii raion, 1 Sep 1986, *N. Shvedchikova* *s.n.* (MW); Primorsky Krai, Terneiskii raion, 8 Aug 1990, *I. Tatarenko* *s.n.* (MW); Sakhalin Oblast, 12 Aug 1990, *V.M. Dvorakovskaya s.n.* (MHA); Primorsky Krai, Terneiskii raion, 21 Jul 1995, *I. Tatarenko 1482* (MW); Sakhalin Oblast, Uzhno-Kuril'skii raion, 6 Aug 1995, *I. Tatarenko s.n.* (MW); Sakhalin Oblast, Uzhno-Kuril'skii raion, 3 Aug 1995, *I. Tatarenko* *1482* *s.n.* (MW); Kamtchatatca, Mt. Mayak, 150–800 m, 31 Jul 2002, *B.Y. sun, D.O. Lim, T.K. An 2487* (2 sheets) (KH); Primorsky Krai, Lazovskii raion, 11 Aug 2007, *S.V. Dudov 1-018* (MW); Khabarovsk Krai, 12 Aug, *N.I. Shaga 58* (MHA); Sakhalin Oblast, t.p.gr. Uzhno-Sahalinsk, 28 Jul 2014, *K.A. Korznikov 1482* (MW); Sakhalin Oblast, Smirnyhovskii raion, 21 Sep 2015, *D.A. Krivenko 42476* (MHA); same locality, 21 Sep 2015, *D.A. Krivenko 42476* (MW); Sakhalin Oblast, Smirnyhovskii raion, *s.n.* (MW); Siberia, Chukotka & Kamchatka, 25 Jul 1967, *A. Khokhryakov 2517* (MHA); Russian Far East, Primorsky Krai, 14 Jul 2016, *E.L.* Zheleznaya *s.n.* (MHA); Siberia, Russian Far East, Primorsky Krai, 459 m, 16 Jul 2016, *E.L. Zheleznaya s.n.* (MHA); Kamchatka Krai, Bystrinskii raion, 288 m, 18 Sep 2017, *V.V. Buryy BV2020-015*1 (MW); Khabarovsk Krai, Komsomol'skii raion, 176 m, 1 Sep 2019, *V. Chepinoga 33934* (IRKU).

**Japan.** **Hondo:** Kanagawa, Fujisawa City, Kugenuma, 1,981 m, 7 Jun 1914, *T. Makino s.n*. (KH); Kyoto, Shinshu (Currently Nagano Prefecture), 26 Jul 1928, *M. Inagaki s.n.* (NAS); 5 Aug 1907, Kyoto, *Shimadzu Corporation, Specimen Department 434* (PE); Musashi, Saitama, Chichibu-gun, Ogano-machi, Sakamoto, Mt. Futago 500–900 m, 16 Jul 1949, *M. Furuse 20894* (PE); Nagano, Siwa-gun, Fujimi-choo, Nishi-dake Mt. Tatsu, 1,700 m, 12 Sep 1951, *M. Furuse 24374* (PE); Kai, Minami-tsuru-gun, Nishi-kaysura-mura, Mt. Mitsutooge, 1,500 m, 3 Sep 1954, *M. Furuse 28111* (PE); Shizuoka, Uruga, Abegun, Igawa-mura, Mt. Semmai-dake, 2,400–2,800 m, 28 Aug 1958, *M. Furuse 34395* (PE); Shinano, Kami-ina-gun, Miya-mura, Summit of Mt. Shiraiqa, 2,200 m, 12 Aug, 1954, *M. Furuse 27884* (PE); Chiba, Kadzusa, Chyppaei-gun, Yaysumi-mura, 10 Aug 1959, *M. Furuse 35959* (PE); Nagano, Kami-ina-gun, to Zizho-dake Mt, Senjoo-dake hase-mura, 2,370 m, 2, Sep 1979, *M. Furuse 14013* (PE); Tochigi, Shimotsuke, Tokorono Nikko-shi, Mt. Ogura, 650 m, 9 Aug 1988, *M. Furuse 54521* (PE); Nagano, Shinano, Todai Hase-mura Kami-ina-gun, Vicinity, and Maku-iwa, 1,800m, 27 Jul 1989, *M. Furuse s.n.* (PE). **Hokkaido:** Hokkaido, Mt. Rishiri Kitami, 150 m, 12, Aug, 1952, *M. Furuse 25406* (PE); Hidaka Yezo, Samani-gun, Yobetsu-mura, 25 Jul 1955, *M. Furuse 29398* (PE); Hidaka Yezo, Samani-gun, Mt. Apoi, 200 m, 28 Jul 1955, *M. Furuse 29511* (2 sheets) (PE); Hidaka Yezo, Samani-gun, Samani-mura, Mt. Apoi, 500 m, 30 Jul 1957, *M. Furuse 32739* (PE); Kitami,kafuka-mura Isl. Rebun, Kafukai-tooge, 50–170 m, 14 Aug 1975, *M. Furuse 9941* (PE); Teshio, Teshio-gun, Horonobe-choo, Toikanbetsu, Nuporo maporo-river, 30–50 m, 10 Aug 1976, *M. Furuse 11557* (PE).
